# Intra-Tumoral Expression of SLC7A11 Is Associated with Immune Microenvironment, Drug Resistance, and Prognosis in Cancers: A Pan-Cancer Analysis

**DOI:** 10.3389/fgene.2021.770857

**Published:** 2021-12-02

**Authors:** Jiajun He, Hongjian Ding, Huaqing Li, Zhiyu Pan, Qian Chen

**Affiliations:** Department of General Surgery, Minhang Hospital, Fudan University, Shanghai, China

**Keywords:** ferroptosis, SLC7A11, pan-cancer, immune microenvironment, drug resistance

## Abstract

While many anti-cancer modalities have shown potent efficacy in clinical practices, cancer prevention, timely detection, and effective treatment are still challenging. As a newly recognized iron-dependent cell death mechanism characterized by excessive generation of lipid peroxidation, ferroptosis is regarded as a potent weapon in clearing cancer cells. The cystine/glutamate antiporter solute carrier family 7 member 11 (SLC7A11) is the core target for ferroptosis regulation, the overexpression of which dictates downregulated sensitivity to ferroptosis in cancer cells. Hence, we elaborated the pan-cancer level bioinformatic study and systematically elucidated the role of intra-tumoral expression of SLC7A11 in the survival of cancer patients and potential immunotherapeutic response. Specifically, 25/27 (92.6%) cancers were featured with upregulated SLC7A11 expression, where SLC7A11 overexpression is a risk factor for worse overall survival in 8 cancers. We also validated SLC7A11 expression in multiple pancreatic cancer cell lines *in vitro* and found that it was upregulated in most pancreatic cancer cell lines (*p* < 0.05). Single-cell sequencing method revealed the SLC7A11 was majorly expressed in cancer cells and mononuclear cells. To further explore the function of SLC7A11 in cancer progression, we analyzed the influence on cell proliferation after the knockdown or knockout of SLC7A11 by either CRISPR or RNAi methods. Besides, the association between SLC7A11 and drug resistance was characterized using bioinformatic approaches as well. We also analyzed the association between the expression of SLC7A11 in multi-omics level and the intra-tumoral infiltration of immune cells based on cell annotation algorithms. Moreover, the relationship between SLC7A11 and the expression of MHC, immune stimulators, immune inhibitors as well as the response to immunotherapy was investigated. In addition, the SLC7A11 expression in colon adenocarcinoma, uterine corpus endometrial carcinoma, and stomach adenocarcinoma (STAD) is also positively associated with microsatellite instability and that in head and neck squamous cell carcinoma, STAD, and prostate adenocarcinoma is positively associated with neoantigen level, which further revealed the potential relationship between SLC7A11 and immunotherapeutic response.

## Introduction

Cancer is the leading cause of human deaths in the world, which produces serious economic burdens both in developed and developing countries (Wu et al., 2019). Although many anti-cancer modalities, such as neoadjuvant chemotherapy and immunotherapy, have shown potent efficacy in clinical practice, cancer prevention, early detection, and effective treatment are still challenging in most cases (O'Donnell et al., 2019; Yang, 2015; Burotto et al., 2019). Hence, finding a more effective strategy to treat cancers is necessary and urgent.

Ferroptosis, a newly recognized iron-dependent cell death mechanism, is characterized by excessive generation of lipid peroxidation (Tang et al., 2020). Recently, an increasing number of studies reported that ferroptosis is involved in many pathophysiological conditions, including cardiovascular diseases, neurodegenerative diseases, and cancers (Jeong et al., 2017; Liang and Zhang, 2019; Mou et al., 2019). Many studies have reported that ferroptosis-induced cell death is an effective approach in killing cancer cells through reactive oxygen species (ROS) accumulation in cells, although its clinical benefits still need clinical trials for verification (Friedmann Angeli et al., 2019; Hassannia et al., 2019). An *in vivo* study showed that inducing tumor-selective ferroptosis via deletion of a system xC-subunit (SLC7A11) was dramatically contributing to the inhibition of the growth of pancreatic ductal adenocarcinoma, which is one of the most lethal solid organ malignancies (Bai et al., 2018). In addition, Wang et al. demonstrated that PD-1-based immunotherapy combined with ferroptosis induction has a synergistic effect compared with single treatment. Given the low response rate of immunotherapy, ferroptosis induction may be a potent adjuvant modality in anti-cancer immunotherapy (Wang et al., 2019).

As a nutrient transporter frequently overexpressed in human malignancies, SLC7A11 is the cystine/glutamate antiporter solute carrier family 7 member 11 (SLC7A11; also known as xCT) (Sehm et al., 2016; Liu et al., 2019). SLC7A11 could promote cystine uptake and glutathione biosynthesis, leading to protection from oxidative stress and ferroptotic cell death (Sehm et al., 2016; Liu et al., 2019). Depleting SLC7A11 dramatically decreased glutathione concentrations and triggered ferroptosis. A study using genetically engineered mice with SLC7A11 knockout revealed tumor-selective ferroptosis and inhibited the growth of pancreatic cancer (Badgley et al., 2020). In view of its important role in ferroptosis execution, the major reagents that induce ferroptosis are targeted at SLC7A11 like erastin (Bai et al., 2018; Shibata et al., 2019). Several studies have also revealed that SLC7A11 plays vital roles in glutamine metabolism and regulates the glucose and glutamine dependency of cancer cells (Shin et al., 2017). Interestingly, the components in tumor microenvironment could also promote or restrain tumor ferroptotic cell death by influencing the SLC7A11 expression level (Wang et al., 2019; Li et al., 2020; Zhang et al., 2020). CD8^+^ T cells induced ferroptosis in tumor cells through IFN-y-dependent SLC7A11 downregulation (Wang et al., 2019). On the contrary, cancer-associated fibroblasts suppress ferroptosis and promote acquired chemoresistance in gastric cancer through secreting miR-522 (Zhang et al., 2020). Hence, it is significant to investigate the role of SLC7A11 in cancer development. Here, we conducted a bioinformatic study to systematically explore whether the intra-tumoral expression of SLC7A11 is associated with cancer patients’ prognosis and response to immunotherapy.

## Materials and Methods

### The Source of Transcriptome and Clinical Data

The transcriptome data of 33 cancers, including adrenocortical carcinoma (ACC), bladder urothelial carcinoma (BLCA), breast invasive carcinoma (BRCA), cholangiocarcinoma (CHOL), colon adenocarcinoma (COAD), cervical squamous cell carcinoma and endocervical adenocarcinoma (CSEA), lymphoid neoplasm diffuse large B-cell lymphoma (DLBC), esophageal carcinoma (ESCA), glioblastoma multiforme (GBM), head-and-neck squamous cell carcinoma (HNSC), kidney chromophore (KICH), kidney renal clear cell carcinoma (KIRC), kidney renal papillary cell carcinoma (KIRP), acute myeloid leukemia (LAML), brain lower grade glioma (LGG), liver hepatocellular carcinoma (LIHC), lung squamous cell carcinoma (LUSC), lung adenocarcinoma (LUAD), mesothelioma (MESO), ovarian serous cystadenocarcinoma (OV), pheochromocytoma and paraganglioma (PCPG), pancreatic adenocarcinoma (PAAD), prostate adenocarcinoma (PRAD), rectum adenocarcinoma (READ), sarcoma (SARC), skin cutaneous melanoma (SKCM), testicular germ cell tumor (TGCT), thyroid carcinoma (THCA), stomach adenocarcinoma (STAD), thymoma (THYM), uterine corpus endometrial carcinoma (UCEC), uterine carcinosarcoma (UCS), and uveal melanoma (UVM), were downloaded from The Cancer Genome Atlas (TCGA). We merged the transcriptome data of normal pancreas in The Genotype Tissue Expression (GTEx), which is a comprehensive public resource to study tissue-specific gene expression, with TCGA cohort given that the latter lacks normal samples for control. Besides, the expression of SLC7A11 is also evaluated in distinct cancer cell lines through Cancer Cell Line Encyclopedia (CCLE) (https://portals.broadinstitute.org/ccle/data). Fragments per kilobase million (FPKM) was selected as the data format for following calculation. The patients’ clinical information, including overall survival (OS), disease-specific survival (DSS), disease-free interval (DFI), and progression-free interval (PFI), was also downloaded from TCGA and merged with transcriptome data.

### Bioinformatic and Statistical Analysis

A univariate Cox regression analysis was applied to identify the association between SLC7A11 expression and OS, DSS, DFI, and PFI across 33 cancers. Hazard ratio (HR) was used to evaluate the magnitude of association with R package “survival” (version 3.1-8). Kaplan‐Meier survival curve was depicted to visualize those associations with statistical significance.

Optimum cutoff value was determined dependent of the largest Youden index. R Package “Estimate” (version 1.013) was used to estimate the proportion of immune and stromal cells in malignant tumor tissues from transcriptome data. Specifically, we introduced “immune score” to assess the proportion of immune cells and “stromal score” to assess the proportion of stromal cells. Estimate score is equal to the sum of immune and stromal scores. Pearson correlation coefficient (r) was used to evaluate the strength of association between SLC7A11 expression and immune/stromal/estimate scores.

The expression level of 47 immune checkpoint genes was extracted from transcriptome data of each cancer. The co-expression association was also calculated using Pearson correlation coefficient and visualized as a heatmap. The infiltration of six common immune cells, CD8^+^ T cells, CD4^+^ T cells, B cells, macrophages, neutrophils, and dendritic cells, was evaluated using Tumor Immune Estimation Resource (TIMER) database. Tumor mutation burden (TMB) is defined as the total number of somatic gene coding errors, base substitutions, and gene insertions or deletions detected per million bases. We calculated the TMB of each cancer sample based on the exome sequencing data from TCGA (VarScan2). MSI (microsatellite instability) referred to the molecular fingerprint of a deficient mismatch repair system. We referred to previous studies for summarized MSI data across distinct cancers (Hause et al., 2016; Yang et al., 2019). Neoantigens are encoded by mutated genes of tumor cells, which are mainly new abnormal proteins produced by gene point mutation, deletion mutation, and gene fusion that are different from proteins expressed by normal cells. Neoantigen level was obtained from a previously published study (Rooney et al., 2015). The correlation between TMB/MSI/neoantigen and SLC7A11 expression was calculated using Pearson correlation coefficient and visualized as radar plots. Similarly, the association between SLC7A11 expression and the expression level of DNA repair-related regulators 17 and methyltransferases were also evaluated by Pearson correlation coefficient.

Gene set enrichment analysis (GSEA) was performed to identify which pathways are varied along with the differential expression of SLC7A11. The top five enriched pathways were showcased according to the ranking of normalized enrichment score. The results in this study were seen statistically significant when *p* value is less than 0.05.

To analyze the association between SLC7A11 expression and drug resistance in pan-cancer landscape, we first used RNAactDrug database (http://bio-bigdata.hrbmu.edu.cn/RNAactDrug). A total of 562 FDA-approved drugs were analyzed using three common methods, including CellMiner, GDSC, and CCLE.

To present the single-cell transcriptomic expression of SLC7A11, we applied TISCH method to analyze the expression pattern of SLC7A11 in different types of cells in tumor microenvironment (http://tisch.comp-genomics.org/).

We also analyzed the correlation between the methylation, mRNA abundance, and copy number of SLC7A11 and immune infiltration, MHC, and immune stimulators and inhibitors in multiple cancers (http://cis.hku.hk/TISIDB/). To further study the relationship between SLC7A11 expression and T-cell dysfunction and immunotherapeutic response, we turn to TIDE algorithm and performed relevant analysis (http://tide.dfci.harvard.edu/).

### *In Vitro* Validation

The human pancreatic cancer cell line HPDE, BxPC-3, AsPC-1, Capan-1, Panc-1, SW1990, and Mia-Paca2 were obtained from the American Type Culture Collection. Capan-1 cells were cultured in Iscove’s modified Dulbecco’s medium with 10% fetal bovine serum. Panc-1, SW1990, and Mia-Paca2 were cultured in Dulbecco’s modified eagle medium (DMEM). BxPC-3 and AsPC-1 were cultured in Gibco Roswell Park Memorial Institute (RPMI) 1640 Medium. Then, RNA was extracted from cell lines using SteadyPure Universal RNA Extraction Kit (AG21017, China). Quantitative real-time PCR was performed using SYBR green (Qiagen, USA).

To analyze the influence on cell proliferation following by SLC7A11 knockout, we applied DepMap tool (https://depmap.org/) to assess the CERES value for SLC7A11 in cell lines in pan-cancer level. Notably, a smaller CERES value reflected the stronger ability to promote cell proliferation *in vitro*.

### Small Interfering RNA-Mediated Knockdown and CCK-8 Assay

The cells (2 × 10^5^) were seeded in a six-well culture plate. After resting overnight, Lipofectamine 3000 reagent (Invitrogen, Thermo Fisher Scientific, Carlsbad, CA, USA) and the indicated concentration of siRNA were added in serum-free DMEM. The complex was added to antibiotic-free medium. After 48 h, the cells were collected for further experiments. Cell relative viability was determined daily using CCK-8 (Beyotime: C0037) based on the manufacturer’s instructions.

## Results

### The Differential Expression of SLC7A11 in Distinct Cancer Cell Lines, Normal and Tumor Samples Within Bulk or Single-Cell Transcriptomic Landscape

**FIGURE 1:**
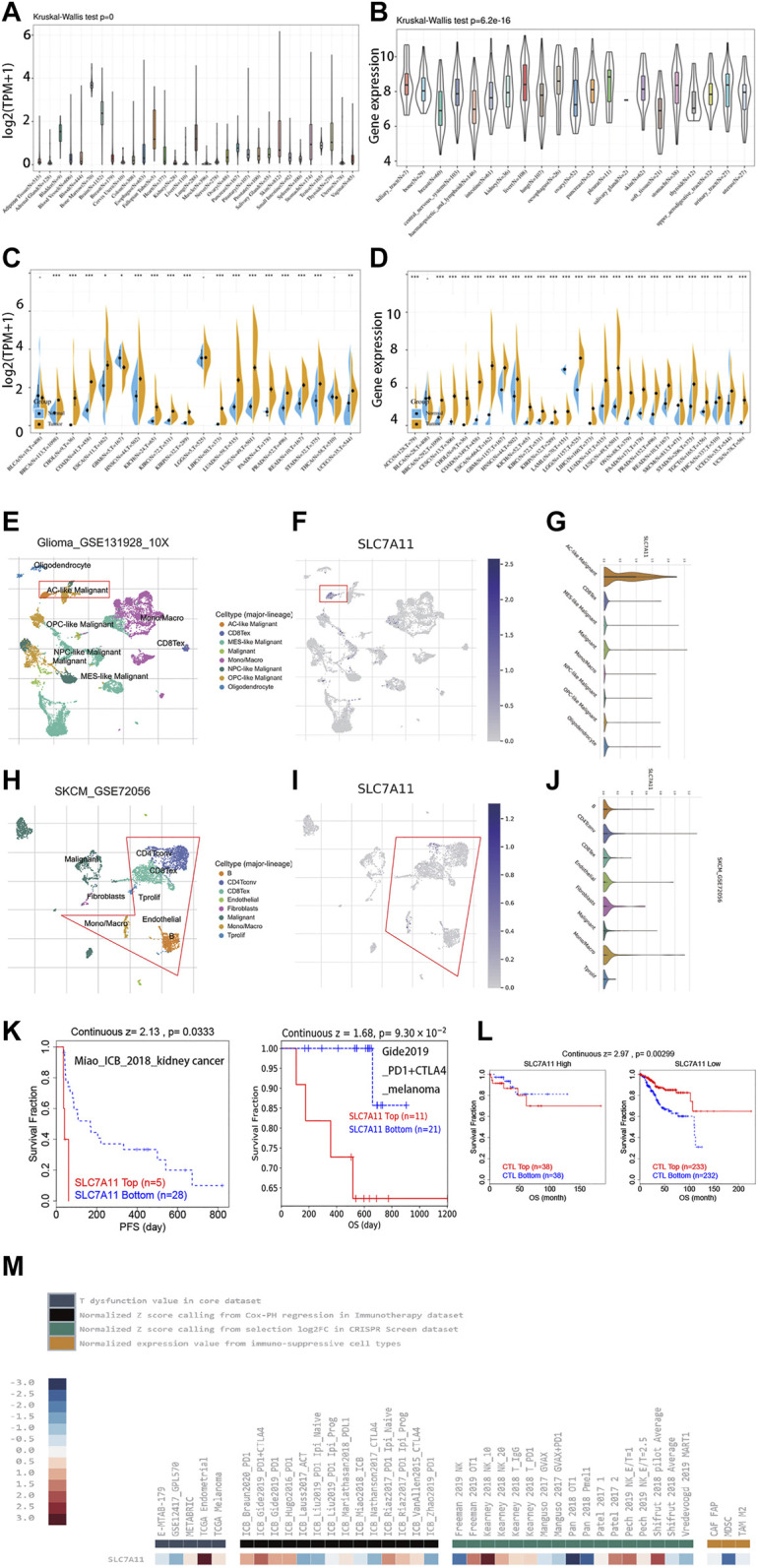
The differential expression of SLC7A11 in distinct cancers. **(A)** The expression level of SLC7A11 in 33 normal organs. **(B)** The expression level of SLC7A11 in 21 cancer cell lines. **(C)** Investigation of the differential expression of SLC7A11 between cancers and normal tissues using TCGA data. **(D)** Investigation of the differential expression of SLC7A11 between cancers and normal tissues using the data from TCGA combined with GTEx. **(E–J)** Single-cell analysis and TIDE algorithms revealing the expression distribution and immunosuppressive characteristic of SLC7A11 in cell clusters. **(K)** Immunotherapy prolonged the survival expectancy only in patients with lower SLC7A11 expression. **(L)** SLC7A11 is associated with increased CTL cytotoxicity based on TIDE algorithm; CTL top referred to samples with higher CTL infiltration, while CTL bottom means lower CTL infiltration. The cut-off value to distinguish high and low CTL infiltration is just dependent on the Cox-PH model embedded in TIDE algorithm. **(M)** Comprehensive presentation of the influence on T-dysfunction and immunotherapeutic response associated with SLC7A11 by TIDE algorithm.

The expression level of SLC7A11 is highly inconsistent across 31 normal tissues (Kruskal‐Wallis test *p* < 0.05) (Figure 1A). Notably, SLC7A11 is highly expressed in bone marrow but rarely expressed in adipose tissue, adrenal gland, cervix uteri, kidney, liver, muscle, nerve, and uterus. This phenomenon suggested that not all organs are sensitive to ferroptosis due to the differential expression of SLC7A11. Because SLC7A11 has a low expression in normal tissues, ferroptosis is thought to occur in normal cells and could be a physiologic activity. Then, we further compared the expression level among cancer cell lines (Figure 1B). Interestingly, the landscape of SLC7A11 expression across cancers changed dramatically. Specifically, SLC7A11 has the highest expression level in pleural but rarely expressed in breast, hematopoietic/lymphoid, and soft tissues. Hence, SLC7A11 may experience a transcriptome remodeling during the carcinogenesis. To support our assumption, we further compared the differential expression of SLC7A11 between human normal and tumor tissues using TCGA data (Figure 1C). The results showed that SLC7A11 is overexpressed in 80% (16/20) cancers, which indicated that many cancers may shrink ferroptosis by upregulating SLC7A11 expression. Due to the insufficiency of normal samples in some tumor categories in TCGA, we further incorporated the transcriptome data from GTEx and re-evaluated the differential expression of SLC7A11 in 27 cancers (Figure 1D). The results noted that 92.6% cancers were featured with upregulated SLC7A11 expression, which further supported our hypothesis. We also validated the differential expression of SLC7A11 in distinct cell lines. Compared with normal pancreatic ductal cells, the expression of SLC7A11 was upregulated in most pancreatic cancer cell lines (*p* < 0.05; Supplementary Figure S1).

Single-cell transcriptomic analysis revealed the expression pattern of SLC7A11 in different cell types (Figures 1E–J; Supplementary Figure S2). First, SLC7A11 is rarely expressed in hematologic malignancies (AEL, AML, and ALL). In solid tumors, SLC7A11 was observed to express highly in the malignant cells of glioma, while for SKCM, SLC7A11 was highly expressed in immune cells and moderately expressed in malignant and stromal cells. For STAD and UCEC, SLC7A11 was more preferred to express in stromal cells. In addition, immunotherapy may only function in patients with lower SLC7A11 expression (Figure 1K). We also presented the comprehensive landscape for the relationship between SLC7A11 and T-dysfunction or immunotherapeutic response in the core cohorts in TIDE database (Figures 1L,M).

### The Correlation Between Intra-Tumoral SLC7A11 Expression and Patients’ Overall Survival, Disease-Specific Survival, Disease-Free Interval, and Progression-Free Interval

**FIGURE 2:**
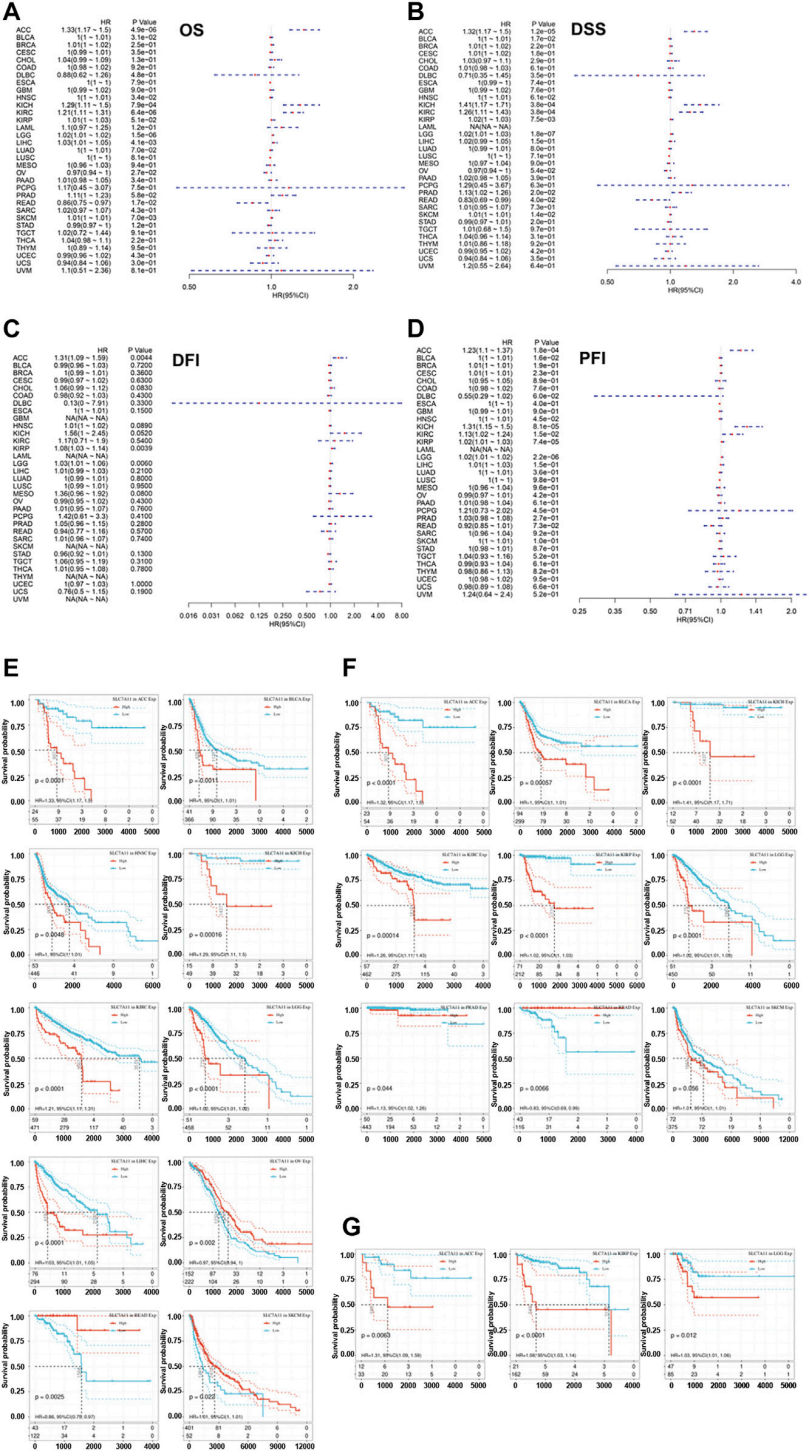
Correlation of the expression of SLC7A11 with the prognosis of distinct cancers. **(A)** The association between SLC7A11 level and overall survival of cancers. **(B)** The association between SLC7A11 level and disease-specific survival of cancers. **(C)** The association between SLC7A11 level and disease-free interval of cancers. **(D)** The association between SLC7A11 level and progression-free interval of cancers. **(E–G)** The survival curve revealed that SLC7A11 is significantly associated with the prognosis of several cancers.

Given the obviously differential expression of SLC7A11 observed between tumor and normal samples, we further investigate whether SLC7A11 expression influences patients’ prognosis. OS, DSS, DFI, and PFI were selected as the indicators to reflect patients’ prognosis. Overexpression of SLC7A11 was identified as a risk factor for worse OS in eight cancers (ACC, BLCA, HNSC, KICH, KIRC, LGG, LIHC, and SKCM) (HR > 1, *p* < 0.05). On the contrary, SLC7A11 served as a protective factor for prolonged OS in OV and READ (HR < 1, *p* < 0.05) (Figure 2A and Figure 2E). In addition, SLC7A11 overexpression was an adverse factor for the DSS of patients with eight cancers (ACC, BLCA, KICH, KIRC, KIRP, LGG, PRAD, and SKCM) (HR > 1, *p* < 0.05); however, it was a favorable factor for better DSS in READ (HR < 1, *p* < 0.05) (Figure 2B and Figure 2F). SLC7A11 is negatively associated with longer DFI in ACC, LGG, and KIRP (HR > 1, *p* < 0.05) and better PFI in ACC, BLCA, HNSC, KICH, KIRC, KIRP, and LGG (HR > 1, *p* < 0.05) (Figures 2C,G, respectively). Overall, excessive expression of intra-tumoral SLC7A11 may be an unfavorable factor for patients’ prognosis in several cancers. It is biologically plausible that tumor cells upregulate SLC7A11 expression to shirk ferroptosis execution and further undermine patients’ survival.

### SLC7A11 Barely Influenced Tumor Cell Proliferation *In Vitro*

**FIGURE 3:**
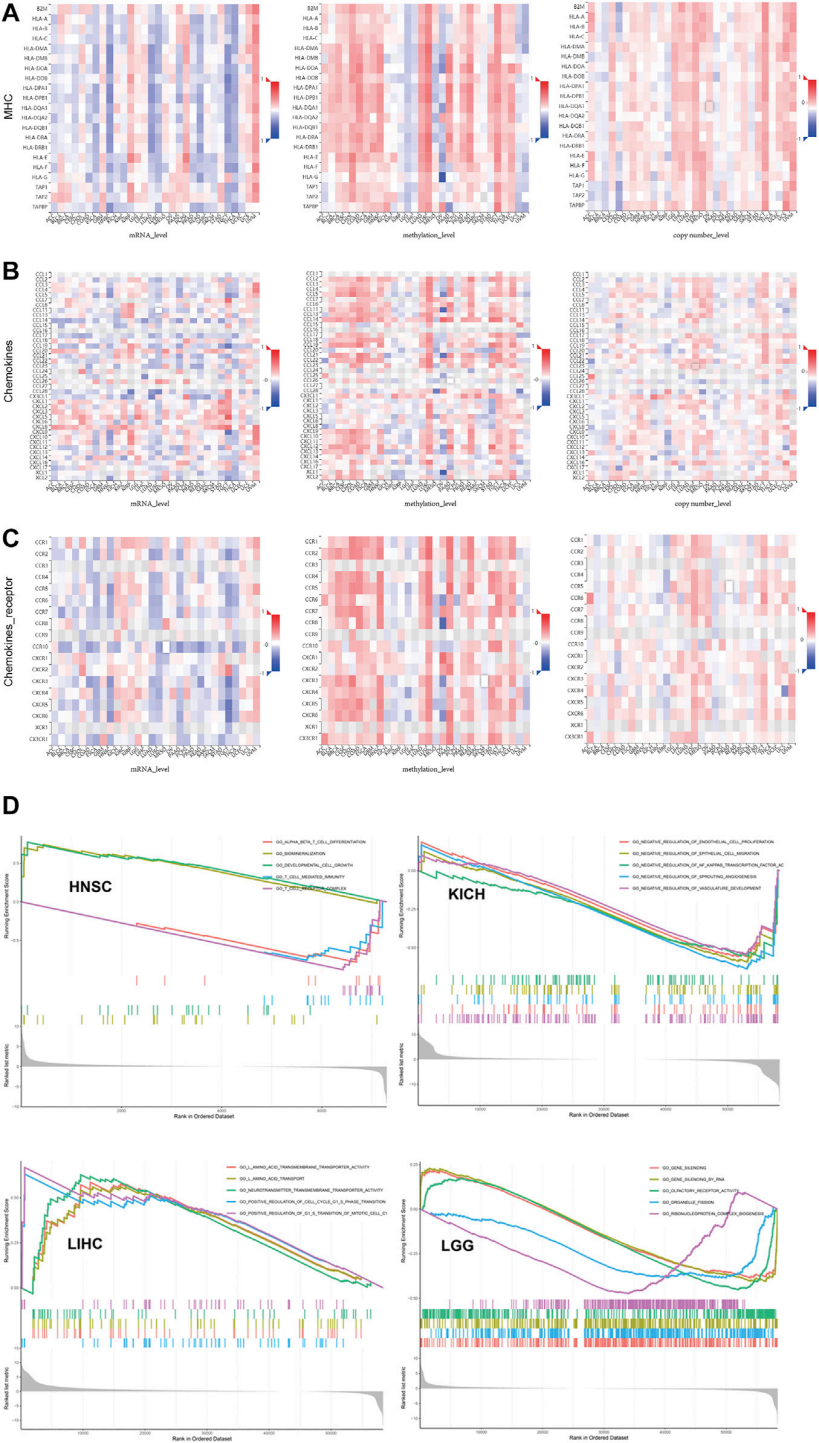
Multi-omics data showed SLC7A11 expression was broadly associated with immune biomarkers but with tumor specificity. **(A)** MHC. **(B)** Chemokines. **(C)** Chemokine receptor. **(D)** GSEA to investigate which signal pathways were altered along with differential expression of SLC7A11 in cancers whose prognosis was associated with SLC7A11.

Given that SLC7A11 was associated with the prognoses in multiple cancers, we studied whether SLC7A11 affected the proliferation of cancer cells. We analyzed the CRISPR- and RNAi-based data in DEPMAP, and the results derived from 989 to 709 cell lines, respectively, showed SLC7A11 knockout or knockdown barely compromised the proliferation ability of all kinds of tumor cells (Figure 3A), which suggested SLC7A11 is associated with the worse prognosis of cancer patients through other mechanisms, tumor microenvironment, for example. To further confirm this finding, we knocked down the expression of SLC7A11 *in vitro* in Panc-1 cell and found SLC7A11 knockdown did not affect the proliferation of pancreatic cancer (Figures 3C,D). We also identified the differentially expressed molecules after SLC7A11 knockdown or knockout, which could be structurally interacted molecules for SLC7A11 (Supplementary Table S1).

We further analyzed the association between SLC7A11 expression and drug sensitivity during cancer treatment using RNAactDrug database. Among 562 FDA-approved drugs, we found the expression of SLC7A11 in mRNA level was significantly positively correlated with six drugs (R > 0.4 and fdr<0.01), including selendale, gelcohol, n6-benzyladenosine-5′-phosphate, 6-benzylthioinosine, (E)-3-(3-((1-(4-fluorobenzyl)-1H-1,2,3-triazol-4-yl)met, kinetin riboside, adenosine, 8-chloro-, cyclic 3′,5′-(hydrogen phosphate), and (E)-1-(benzo[d] (Yang, 2015; Wu et al., 2019)dioxol-5-yl) (Supplementary Table S2). Besides, we investigated the relationship between SLC7A11 and drug resistance in TISIDB, the results of which showed small molecule riluzole, thimerosal, cystine, acetylcysteine, sulfasalazine, and glutamic acid (Figure 3B). Notably, SLC7A11 was a key regulator for the transportation of cystine and glutamic acid, which supported the results yield by analyzing TISIDB tool.

### The Correlation Between SLC7A11 Expression and Immune Cell Infiltration, Immune Score, Stromal Score, and Estimate Score

**FIGURE 4:**
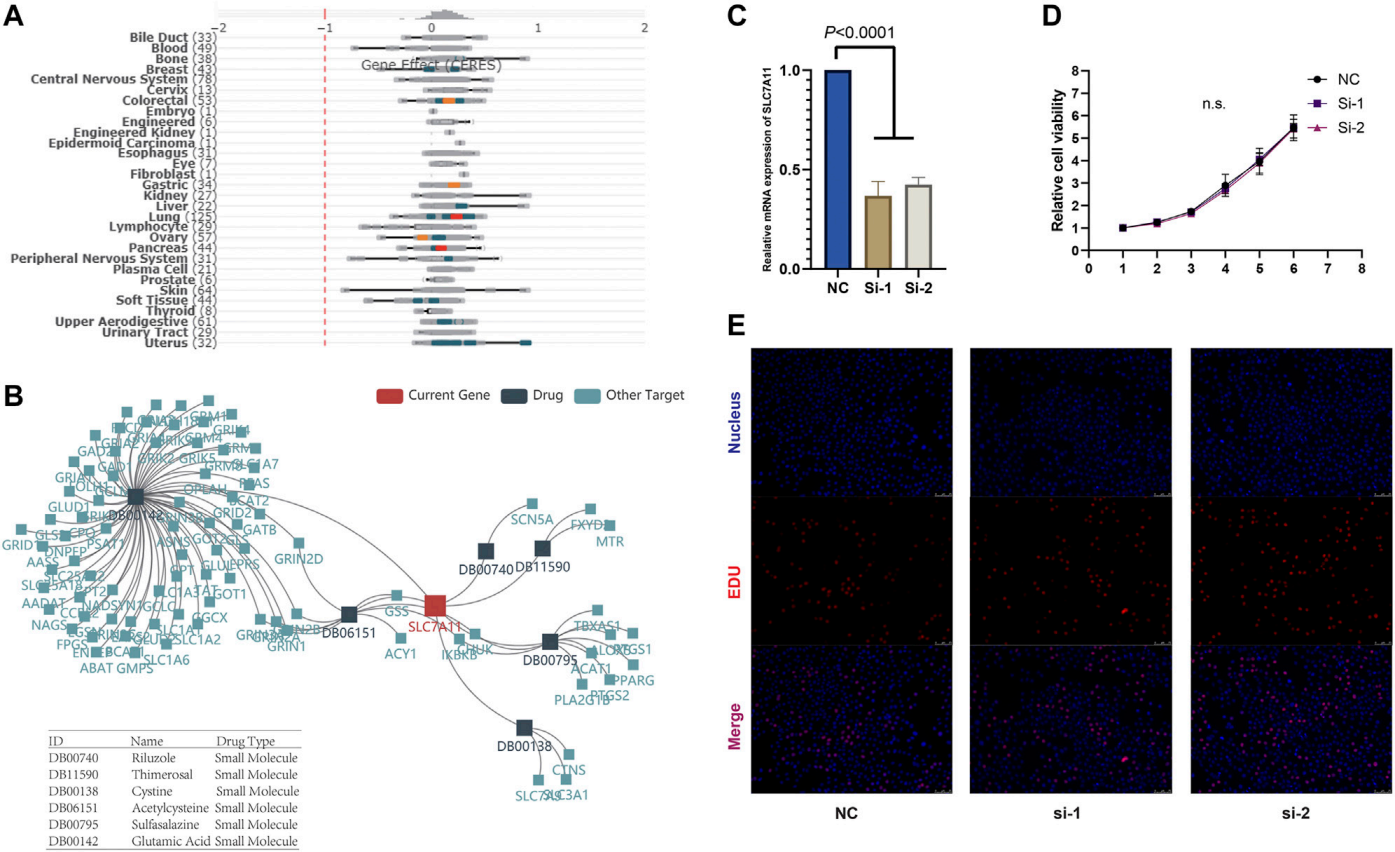
**(A)** SLC7A11 expression barely affects tumor cell proliferation *in vitro*. **(B)** SLC7A11 expression is associated with the sensitivity to multiple drugs. **(C)** Knockdown of SLC7A11. **(D, E)** CCK-8 and EDU analysis shows there are no obvious changes in cell proliferation after SLC7A11 knockdown in panc-1 cell.

Recently, a well-conducted study reported that CD8^+^ T cells could induce ferroptosis in tumor cells via downregulating the SLC7A11 expression (Wang et al., 2019). The combined induction of ferroptosis with immune checkpoint inhibitors has a synergistic effect in anti-tumor therapy. Hence, we speculated SLC7A11 is associated with the immune microenvironment of cancers. The association between SLC7A11 expression level and immune cell infiltration, immune score, stromal score, and estimate score was evaluated using Pearson correlation coefficient (Figure 4A). The results showed that SLC7A11 expression is negatively associated with CD8^+^ T-cell infiltration in seven cancers (DLBC, ESCA, HNSC, LUAD, LUSC, TGCT, and THCA), suggesting the combination of ferroptosis induction and immunotherapy may be suitable in these cancers’ treatment (Supplementary Figure S3). In addition, the association between immune score and SLC7A11 expression is dependent on cancer type. For PAAD, CESC, ESCA, HNSC, LUAD, LUSC, TGCT, and THCA, SLC7A11 expression is negatively associated with immune score. On the contrary, for KIRC, KIRP, LGG, PCGC, THYM, and UVM, SLC7A11 expression is positively associated with the immune score. The relationship between SLC7A11 expression and stromal/estimate score showed a similar trend. We depicted the top three cancers where the correlation coefficient between SLC7A11 expression and immune/stromal/estimate scores is largest as Supplementary Figure S4. Normally, tumor purity is negatively associated with immune/stromal score. Therefore, in such cancers that SLC7A11 is negatively associated with immune/stromal score, SLC7A11 may be expressed more in tumor cells instead of immune/stromal cells. Interestingly, there are major overlaps for cancers that SLC71A11 expression level is negatively associated with immune/stromal score while positively correlated with CD8^+^ T-cell infiltration (CESC, ESCA, HNSC, LUAD, LUSC, TGCT, and THCA). In these cancers, SLC7A11 is assumed to express majorly in tumor cells and may be regulated by immune cells in stroma like CD8^+^ T cells.

### The Association Between SLC7A11 Expression and Potential Response to Immunotherapy Across Distinct Cancers

To optimize the response rate of immunotherapy, in recent years, many clinical trials tried to add chemotherapeutics in regular immunotherapy and observed a synergistic effect. Several studies have reported that chemotherapeutic drugs have the capacity to induce ferroptosis *in vivo* and *ex vivo*. Hence, it is significant to investigate whether there are correlations between SLC7A11 expression and patients’ potential response to immunotherapy in the pan-cancer level. At present, the expression level of immune checkpoint markers, MSI, TMB, and neoantigen were four major indicators for predicting patients’ response to immunotherapy. Here, we analyzed the correlation between SLC7A11 expression and the level of these immunotherapeutic markers (Figure 4B). Two widely studied immune checkpoints, PDCD1 (PD-1) and CD274 (PD-L1), were positively correlated with SLC7A11 expression in LUAD and LGG, suggesting simultaneously targeting SLC7A11 and PD1/L1 could be beneficial in LUAD and LGG. In addition, NRP1, CD276, and VSIR showed obvious positive correlation with SLC7A11 expression in most cancers, although the drugs targeted at these checkpoints have not been applied in clinic. The TMB of STAD, PRAD, PAAD, HNSC, COAD, BRCA, and UCEC is positively correlated with SLC7A11 expression (Figure 4C). Among these cancer types, the SLC7A11 expression in COAD, UCEC, and STAD is also positively associated with MSI (Figure 4D). Besides, the expression of SLC7A11 in STAD and PRAD was positively associated with neoantigen level (Supplementary Figure S5).

**FIGURE 5:**
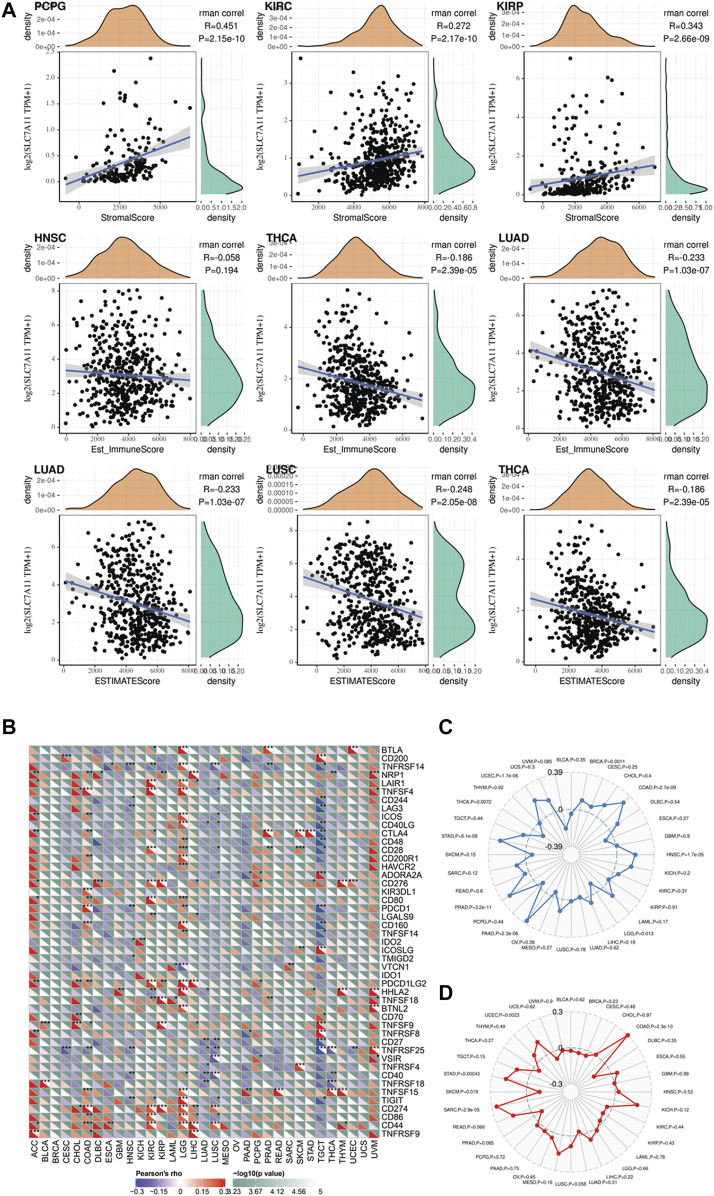
**(A)** The correlation between SLC7A11 expression and immune and stromal score in distinct cancers. **(B–D)** Exploring the potential of SLC7A11 in anti-cancer immunity by evaluating its association with immune checkpoint, tumor mutation burden, and microsatellite instability across distinct cancers.

In addition, we further studied the association between multi-omics level of SLC7A11 and immune variables (Figures 5A–C and Supplementary Figure S6). Overall, mRNA and copy numbers of SCL7A11 were correlated with most molecules involved with MHC, chemokines, and chemokine receptors; however, there was an opposite trend for the correlation between SLC7A11 methylation level and these markers, which is attributed to high methylation enrichment that caused the difficulty in transcription. Furthermore, we analyzed the correlation between SLC7A11 multi-omics expression and immune cell infiltration. The results showed that the relationship between SLC7A11 and each cell subtype was tumor specific. For example, SLC7A11 was negatively associated with activated T cells in most cancer types; however, its mRNA level was positively correlated to infiltrated activated T cells in KIRP and UVM. Accordingly, the methylation of SLC7A11 was negatively associated with activated T cells in KIRP and UVM (Supplementary Figure S6). Besides, the correlation between SLC7A11 and immune stimulators and inhibitors was complicated and tumor specific.

### SLC7A11 Expression Altered in Distinct Molecular and Immune Subtypes

**FIGURE 6:**
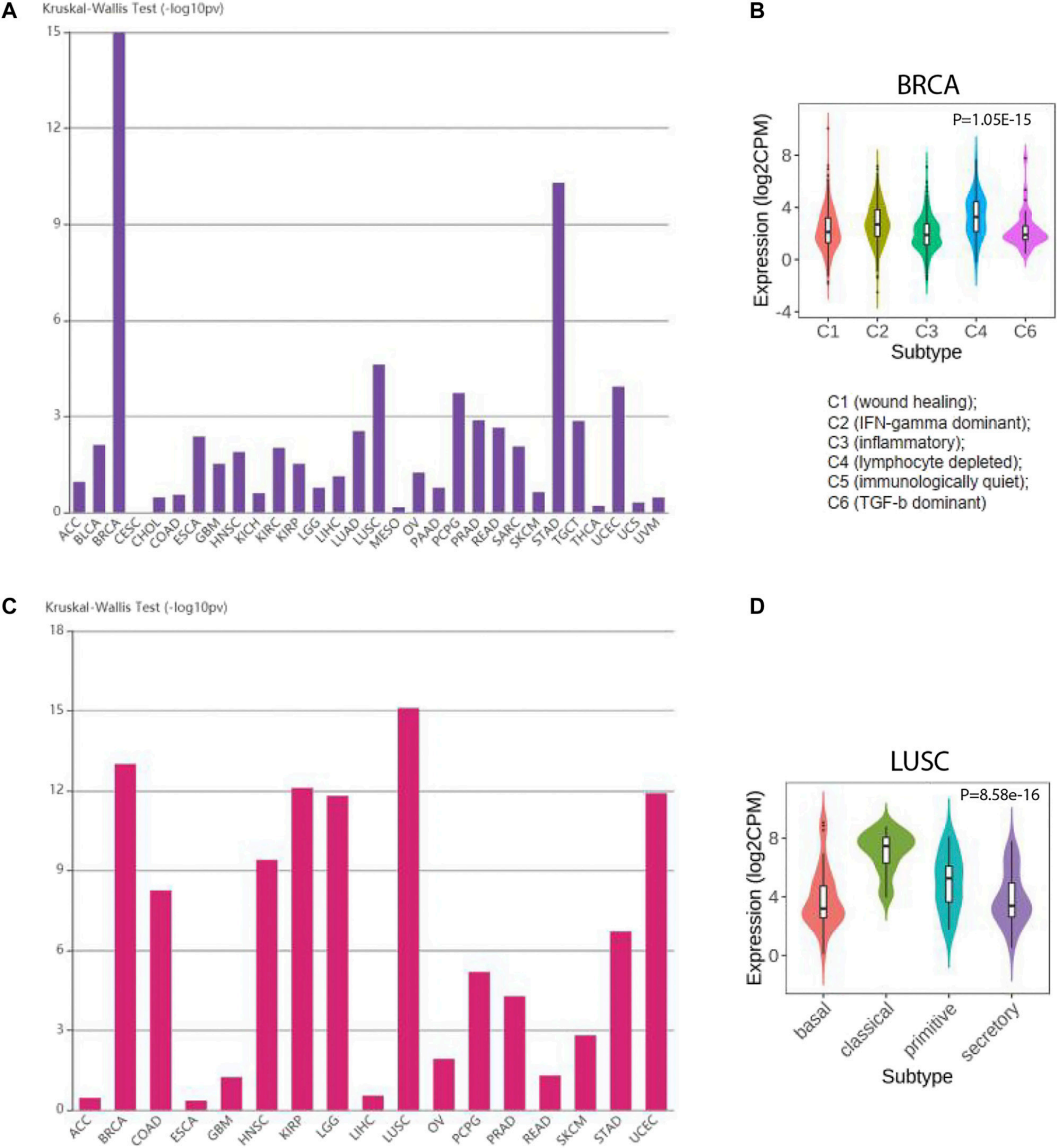
The expression of SLC7A11 is altered in different molecular and immune subtypes of cancers. **(A, B)** SLC7A11 expression in distinct molecular subtypes for cancers. **(C, D)** SLC7A11 expression in distinct immune subtypes for cancers.

In recent years, the development of high-throughput sequencing promoted the molecular and immune subtypes for cancers. Molecular subtypes normally referred to the cancer subtypes based on clustering of differentially expressed genes. Immune subtypes were determined by the differences of immune cell infiltration in tumor microenvironment. Hence, we studied whether SLC7A11 expression was altered in different subtypes. Notably, for immune subtypes, SLC7A11 expression was significantly different in BRCA. SLC7A11 harbors the highest expression in cluster 4 (lymphocyte depleted cluster), which indicated a negative correlation for SLC7A11 expression and lymphocyte infiltration. However, for cluster 3, which was referred to inflammatory subtype, SLC7A11 harbored the lowest expression level. For molecular subtypes, SLC7A11 had the most prominent differential expression in LUSC. Specifically, classic LUSC subtype had the highest expression of SLC7A11, while secretary and basal LUSC were featured with lower SLC7A11 level (Figure 6).

### GSEA Identified the Signal Pathways that Altered Along with Differential Expression of SLC7A11

To further explore the function of SLC7A11 in cancer biology, we performed a GSEA to investigate which signal pathways were altered along with differential expression of SLC7A11 in cancers whose prognosis was associated with SLC7A11 (Figure 5D). We visualized the top five gene sets enriched in SLC7A11-high or SLC7A11-low tumor samples. The results provided some valuable information. First, gene sets associated with T cellmediated immunity, T-cell receptor complex, and T-cell differentiation were enriched in HNSC samples with relatively low expression of SLC7A11, while one gene set associated with cell growth and development was enriched in HNSC samples with higher expression of SLC7A11. Second, the GSEA results in LIHC showed gene sets associated with amino acid transport and transmembrane were enriched in samples with higher SLC7A11, which may be contributed to the molecular function of SLC7A11 self. In addition, gene sets associated with G1-S phase transition and mitotic cell cycle were also enriched in LIHC samples with higher SLC7A11 expression. Third, gene sets that negatively regulate NF-kappaB transcription factor, vascular development, and angiogenesis were enriched in KICH samples with lower expression of SLC7A11. In conclusion, compared with SLC7A11-low tumor samples, cancers with higher SLC7A11 expression demonstrated decreased anti-cancer immunity, enhanced metabolic activity, and promoted cell division.

## Discussion

An increased number of studies have revealed an anti-cancer role of ferroptosis in recent years (Lu et al., 2017; Liang and Zhang, 2019). It is significant to investigate the implication of ferroptosis-related signature in real-world patients’ tumor samples. As a core gene of ferroptosis, SLC7A11 overexpression naturally downregulates the sensitivity for ferroptosis execution (Jeong et al., 2017; Liu et al., 2019). SLC7A11 is the major target for manipulating ferroptosis *in vivo* and *in vitro* at present (Sehm et al., 2016; Xie et al., 2016; Dahlmanns et al., 2017). Hence, it is imperative to explore the expression and clinical relevance of SLC7A11 in cancer samples. This study systematically established the unfavorable role of SLC7A11 for longer survival, decreased new events, and potential response to immunotherapy in several cancers.

Thanks to the advancement of RNA-sequencing methodology, oncologists could develop a more detailed classification for cancers based on the variation in cancer transcriptome (Jeong et al., 2017; Uhlen et al., 2017). Evolutionally, tumor cell acquired unlimited proliferative capacity through the remodeling in a series of signal pathways (Nygren, 2001; Boumahdi and de Sauvage, 2020). The plasticity of transcriptome conferred tumor cells with accumulated resistance for many chemotherapeutics, which dramatically restrained the efficacy of anti-cancer treatment and compromised patients’ survival (Meacham and Morrison, 2013). Mechanistically, most chemotherapeutic drugs worked out via inducing apoptosis in tumor cells (Pérez-Herrero and Fernández-Medarde, 2015). These drugs normally showed promising efficacy at the beginning, while gradually losing their ability after several rounds of administrations, which attributed to the adaptive resistance to apoptosis execution developed in tumor cells (Goldar et al., 2015). Hence, scientists are constantly seeking a potent strategy to kill chemotherapy-resistant tumor cells. In recent years, as a novel concept, ferroptosis is defined as an iron-dependent accumulation of lipid peroxidation products to death (Hirschhorn and Stockwell, 2019). Many studies observed that chemotherapy-resistant tumor models are still sensitive to ferroptosis induction, which may become an effective weapon for the treatment of these malignancies (Liang and Zhang, 2019). Besides, ferroptosis self is also an important mechanism by which chemotherapeutic drugs kill cancer cells (Sun et al., 2016). For example, Sun et al. have demonstrated that the sensitivity to sorafenib in LIHC could be compromised by ferroptosis inhibition (Sun et al., 2016).

Unlike other non-apoptotic cell death, there are no pore-forming proteins specifically functioned in ferroptosis execution (Liang and Zhang, 2019). In this context, identification of biomarkers that reflect ferroptosis sensitivity is of great importance. As one of the most classic biomarkers of ferroptosis, SLC7A11 controls the influx of cysteine and following glutathione biosynthesis, whose overexpression is associated with ferroptostic insensitivity (Jeong et al., 2017; Koppula et al., 2018). However, few studies systematically discussed the prognostic role of SLC7A11 in cancers. In addition, one well-conducted study first proved CD8^+^ T cells could induce the ferroptosis in cancer cells via downregulating the SLC7A11 transcription and concomitant use of immune checkpoint inhibitors could synergistically enhance the anti-cancer capacity (Wang et al., 2019). This inspired the imagination that whether intra-tumoral expression of SLC7A11 influences the immune microenvironment and potential response to immunotherapy. Hence, we elaborated a bioinformatic study in pan-cancer level to systematically explore whether the intra-tumoral expression of SLC7A11 is associated with patients’ survival and its potential value in immunotherapy. Our results revealed SLC7A11 is implicated in the overall survival of several cancers, including ACC, BLCA, HNSC, KICH, KIRC, LGG, LIHC, and SKCM. Except for these cancers, SLC7A11 is associated with the DSS for PARD and KIRP. In this context, future clinical trials that aim to treat cancers by inducing ferroptosis should first consider these cancers. Our study also focused on the relationship between SLC7A11 expression and TMB, MSI, and neoantigen. Notably, STAD is the only cancer type where the SLC7A11 expression is associated with these three markers for response to immunotherapy simultaneously, which highlighted the value for combined treatment of ferroptosis induction with immunotherapy in STAD.

The present study has some strengths to declare. First, ferroptosis induction is a promising anti-cancer strategy in the near future, and our study provided many valuable and integrated suggestions for the selection of appropriate cancer types. Second, most previous studies investigated the anti-cancer function of ferroptosis in cell/animal level. Although these studies provided more detailed mechanism about the molecular pathways, our study may be more reliable because the tumor samples we selected come from human beings. Third, our study analyzed the association between SLC7A11 expression and potential response to immunotherapy in pan-cancer level, which also brought much valuable information. Certainly, this study has several limitations. On one hand, as an *in silico* research, although our study has a large sample number, appropriate external validation may still be warranted. On the other hand, while this study systematically analyzed the role of SLC7A11 in cancers’ prognosis and immune signatures, the underlying mechanism still needs further investigation by future laboratory research.

In conclusion, this pan-cancer level bioinformatic study systematically elucidated the role of intra-tumoral expression of SLC7A11 in the prognoses, drug resistance, and potential immunotherapeutic response of patients with cancer. Future studies are encouraged to develop clinically approved SLC7A11-targeted drugs and organize safe and effective clinical trials.

## Data Availability

The original contributions presented in the study are included in the article/Supplementary Material, further inquiries can be directed to the corresponding authors.
