# Supplementary material for: Intra-Tumoral Expression of SLC7A11 Is Associated with Immune Microenvironment, Drug Resistance, and Prognosis in Cancers: A Pan-Cancer Analysis
Source: Front Genet. 2021 Dec 2;12:770857. doi: 10.3389/fgene.2021.770857 (PMC8687742; doi:10.3389/fgene.2021.770857)

Supplementary figure 1. The expression of SLC7A11 in pancreatic cancer cell-lines compared with normal pancreatic duct epithelium.

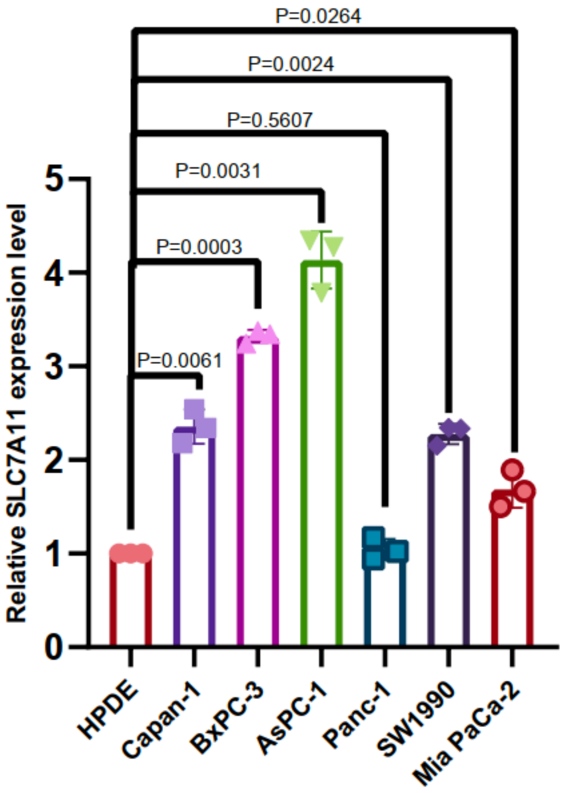

Supplementary figure 2. Single-cell transcriptomic analysis revealed the expression pattern of SLC7A11 in different cell types.

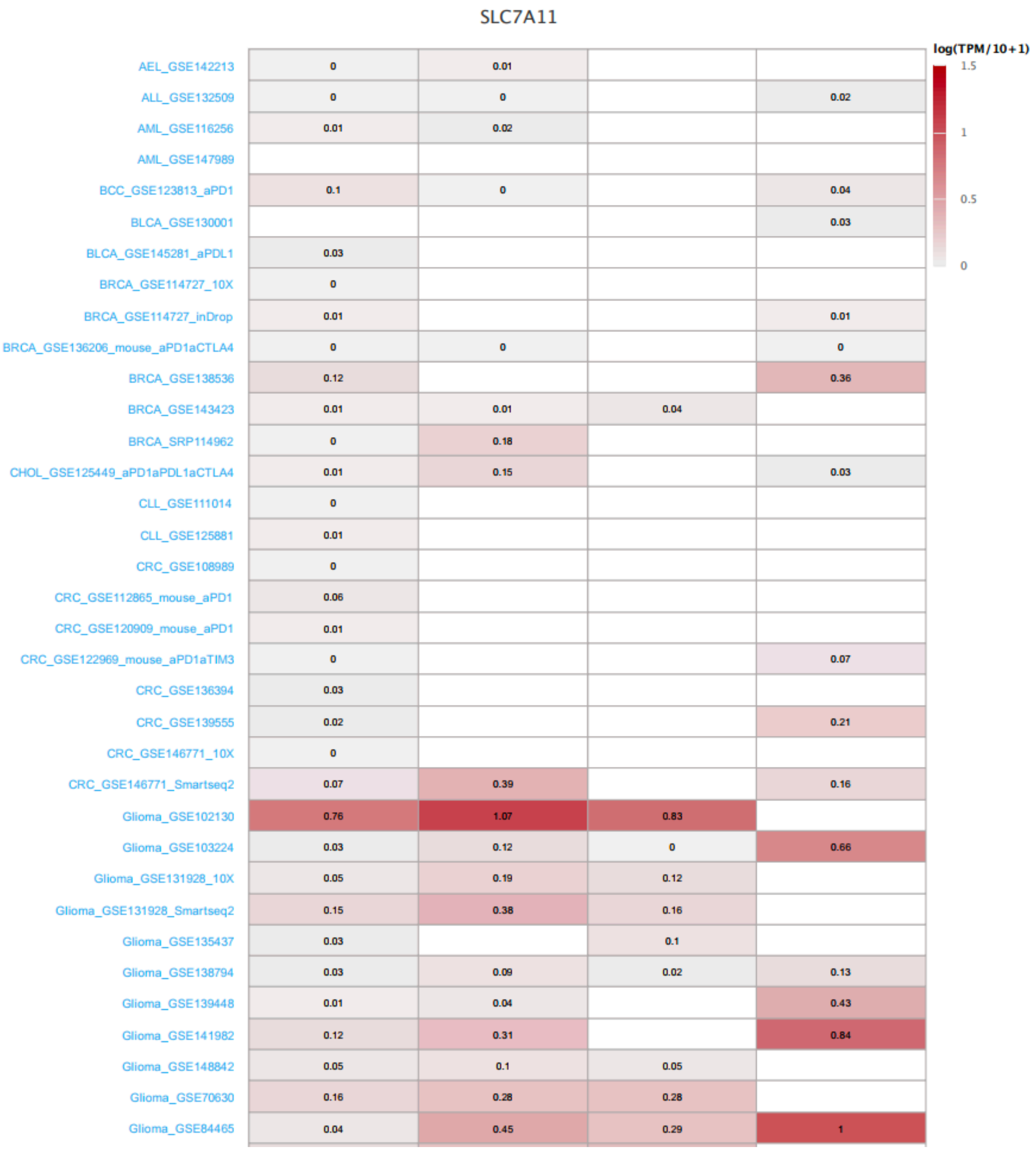

	Immune cells	Malignant cells	Others	Stromal cells
Glioma_GSE89567	0.13	0.28	0.33	
HNSC_GSE103322	0.13	0.87		0.07
HNSC_GSE139324	0.01			
KIRC_GSE111360	0			0
KIRC_GSE139555	0.01			0
KIRC_GSE145281_aPDL1	0.01			
LIHC_GSE125449_aPDL1aCTLA4	0.02	0.01		0.04
LIHC_GSE140228_10X	0			
LIHC_GSE140228_Smartseq2	0.01			
LIHC_GSE98638	0		0	
MB_GSE119926		0.24		
MCC_GSE117988_aPD1aCTLA4	0.01	0.02		0.05
MCC_GSE118056_aPDL1	0.01	0		0.01
MM_GSE117156	0	0.01		
MM_GSE141299		0.01		
NHL_GSE128531	0.03		0	0.01
NSCLC_EMTAB6149	0	0.03	0	0
NSCLC_GSE117570	0	0.11		0.04
NSCLC_GSE127465	0.06	0.27		0.05
NSCLC_GSE131907	0.01		0.07	0.07
NSCLC_GSE139555	0			
NSCLC_GSE143423	0.04	0.14	0.18	0.27
NSCLC_GSE99254	0			
OV_GSE115007	0.05			
OV_GSE118828	0.03	0.21		0.07
PAAD_CRA001160	0.02	0.1	0.01	0.01
PAAD_GSE111672	0.24	0.67	0.28	0.04
PBMC_30K_10X	0			
PBMC_60K_10X	0			
PBMC_8K_10X	0			
SARC_GSE119352_mouse_aPD1aCTLA4	0.13			0
SCC_GSE123813_aPD1	0			
SKCM_GSE115978_aPD1	0.06	0.03		0.06
SKCM_GSE120575_aPD1aCTLA4	0.03			
SKCM_GSE123139	0.01			0.03
SKCM_GSE139249	0			0
SKCM_GSE148190	0		0	
SKCM_GSE72056	0.39	0.23		0.32
STAD_GSE134520	0.05	0.11		0.17
UCEC_GSE139555	0			0.29
UVM_GSE139829	0	0		0.01

Supplementary figure 3. The association between SLC7A11 expression level and immune cell infiltration, immune score, stromal score and estimate score.

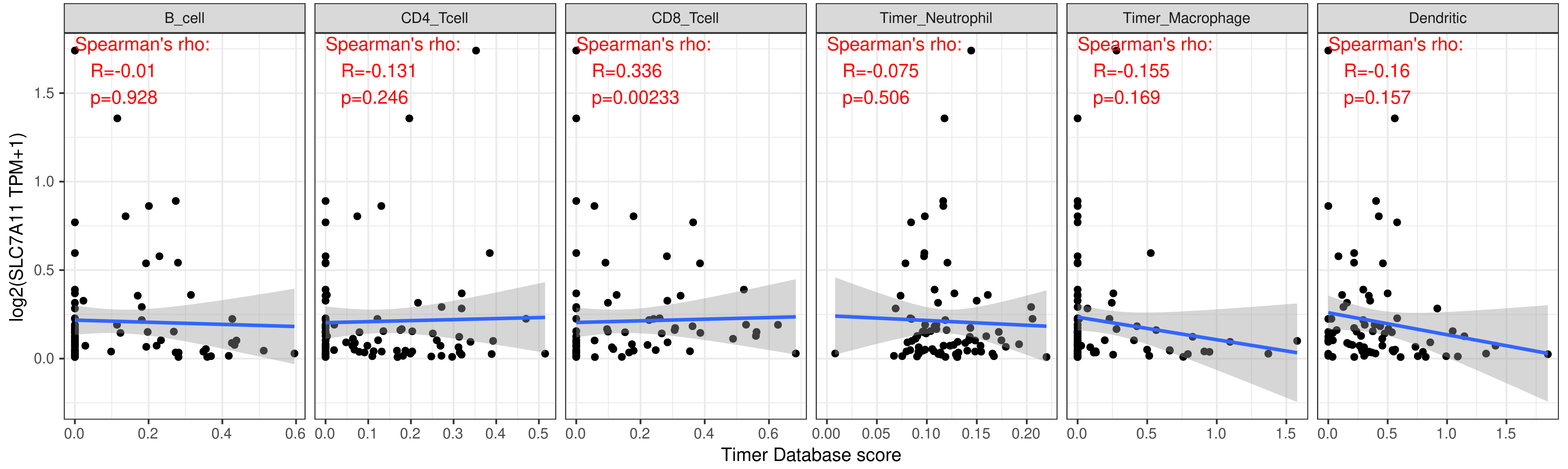
B_cell
CD4_Tcell
CD8_Tcell
Timer_Neutrophil
Timer_Macrophage
Dendritic
Spearman's rho:
R=-0.01
p=0.928
Spearman's rho:
R=-0.131
p=0.246
Spearman's rho:
R=0.336
p=0.00233
Spearman's rho:
R=-0.075
p=0.506
Spearman's rho:
R=-0.155
p=0.169
Spearman's rho:
R=-0.16
p=0.157
log2(SLC7A11 TPM+1)
Timer Database score

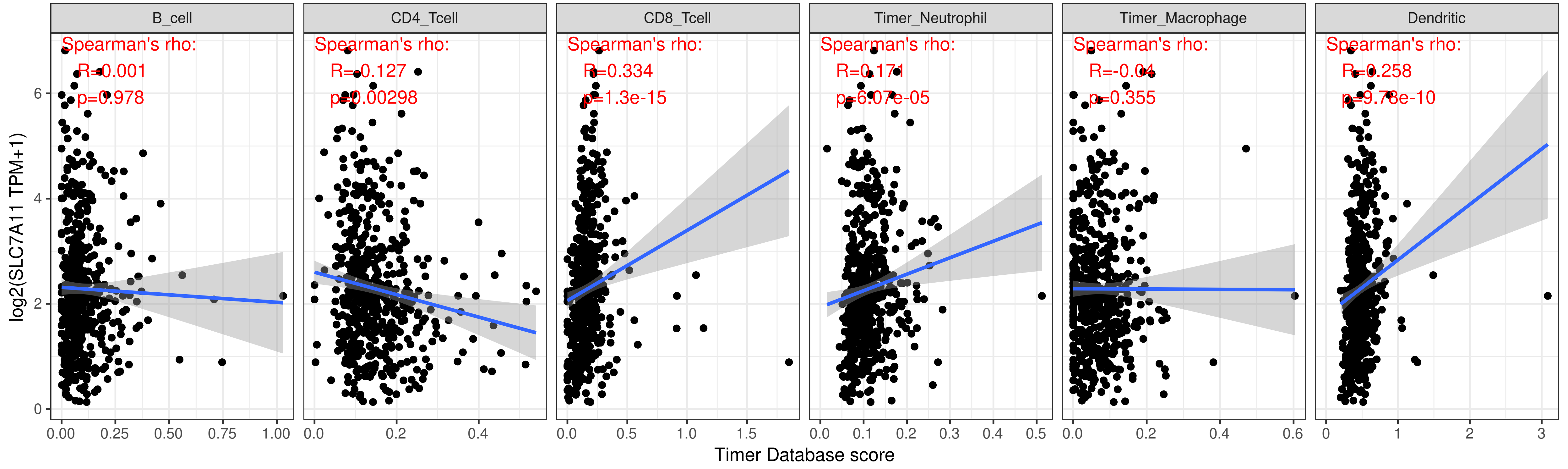

B_cell
CD4_Tcell
CD8_Tcell
Timer_Neutrophil
Timer_Macrophage
Dendritic
Spearman's rho:
R=0.001
p=0.978
Spearman's rho:
R=-0.127
p=0.00298
Spearman's rho:
R=0.334
p=1.3e-15
Spearman's rho:
R=0.171
p=6.07e-05
Spearman's rho:
R=-0.04
p=0.355
Spearman's rho:
R=0.258
p=9.78e-10
log2(SLC7A11 TPM+1)
Timer Database score

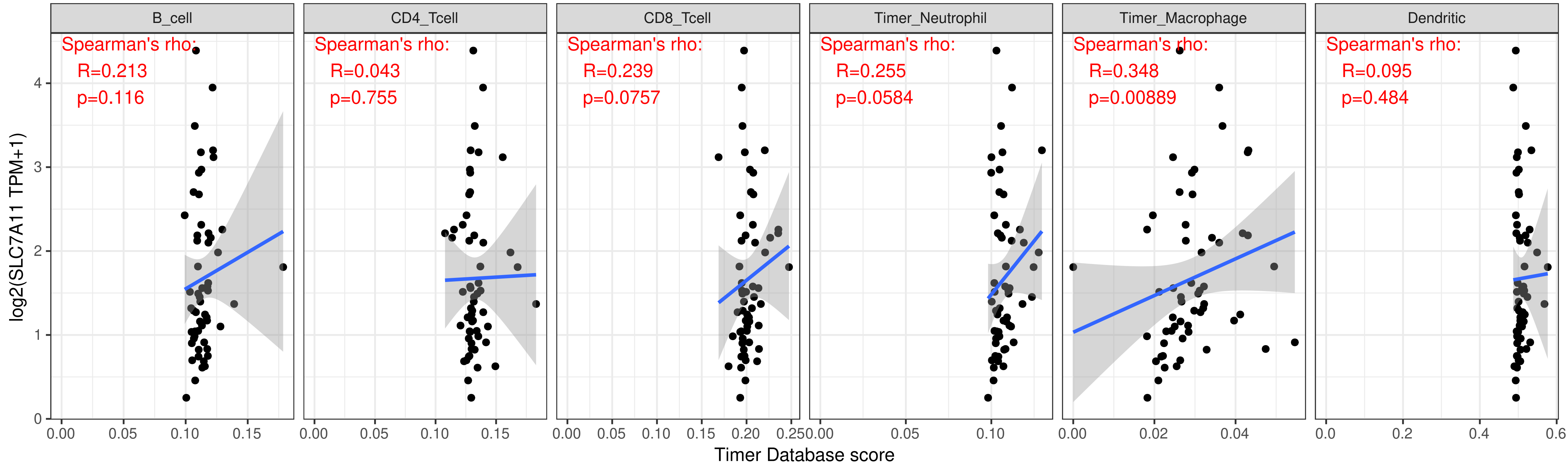
B_cell
Spearman's rho:
R=0.213
p=0.116
CD4_Tcell
Spearman's rho:
R=0.043
p=0.755
CD8_Tcell
Spearman's rho:
R=0.239
p=0.0757
Timer_Neutrophil
Spearman's rho:
R=0.255
p=0.0584
Timer_Macrophage
Spearman's rho:
R=0.348
p=0.00889
Dendritic
Spearman's rho:
R=0.095
p=0.484
log2(SLC7A11 TPM+1)
Timer Database score

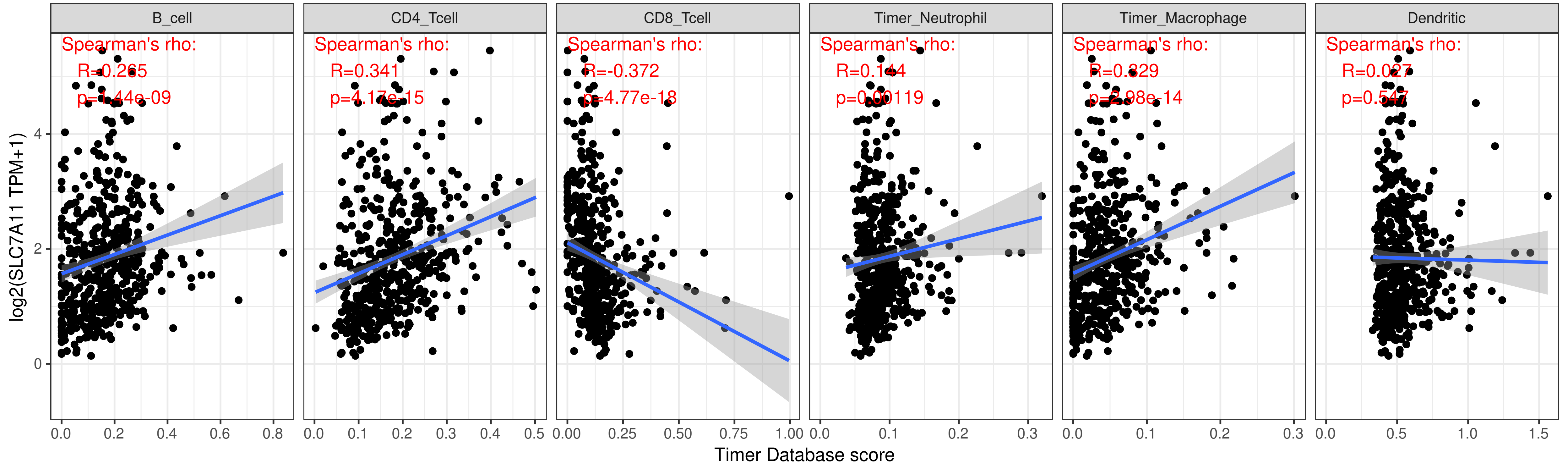
B_cell
Spearman's rho:
R=0.265
p=1.44e-09
CD4_Tcell
Spearman's rho:
R=0.341
p=4.17e-15
CD8_Tcell
Spearman's rho:
R=-0.372
p=4.77e-18
Timer_Neutrophil
Spearman's rho:
R=0.144
p=0.00119
Timer_Macrophage
Spearman's rho:
R=0.329
p=2.98e-14
Dendritic
Spearman's rho:
R=0.027
p=0.547
log2(SLC7A11 TPM+1)
Timer Database score

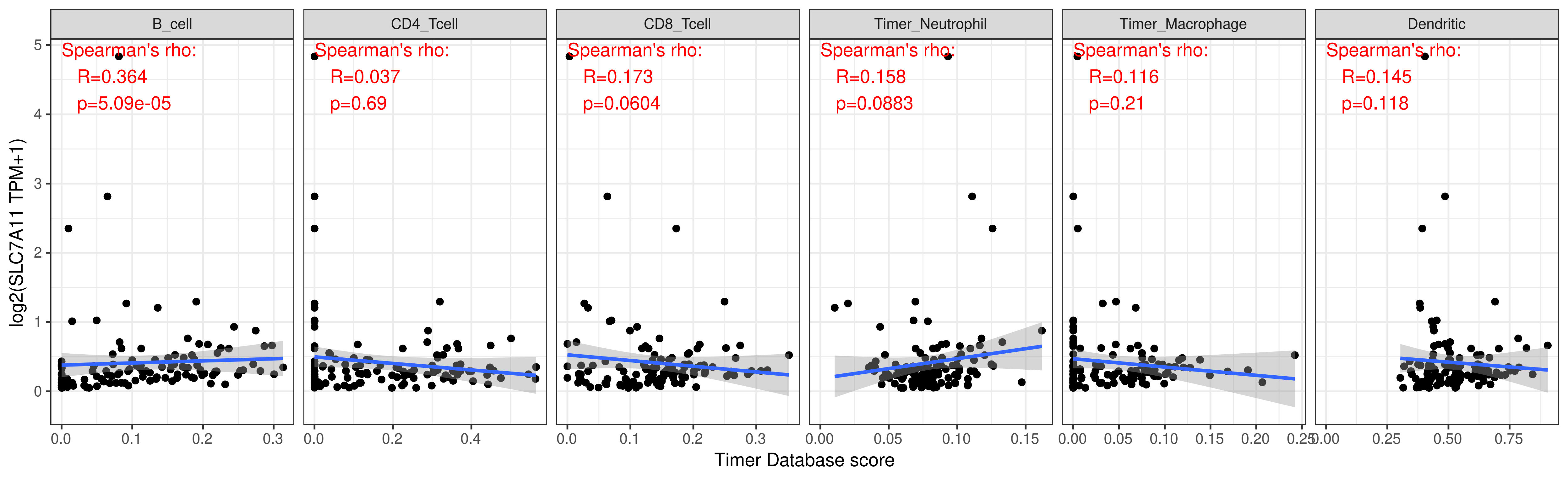
B_cell
Spearman's rho:
R=0.364
p=5.09e-05
CD4_Tcell
Spearman's rho:
R=0.037
p=0.69
CD8_Tcell
Spearman's rho:
R=0.173
p=0.0604
Timer_Neutrophil
Spearman's rho:
R=0.158
p=0.0883
Timer_Macrophage
Spearman's rho:
R=0.116
p=0.21
Dendritic
Spearman's rho:
R=0.145
p=0.118
log2(SLC7A11 TPM+1)
Timer Database score

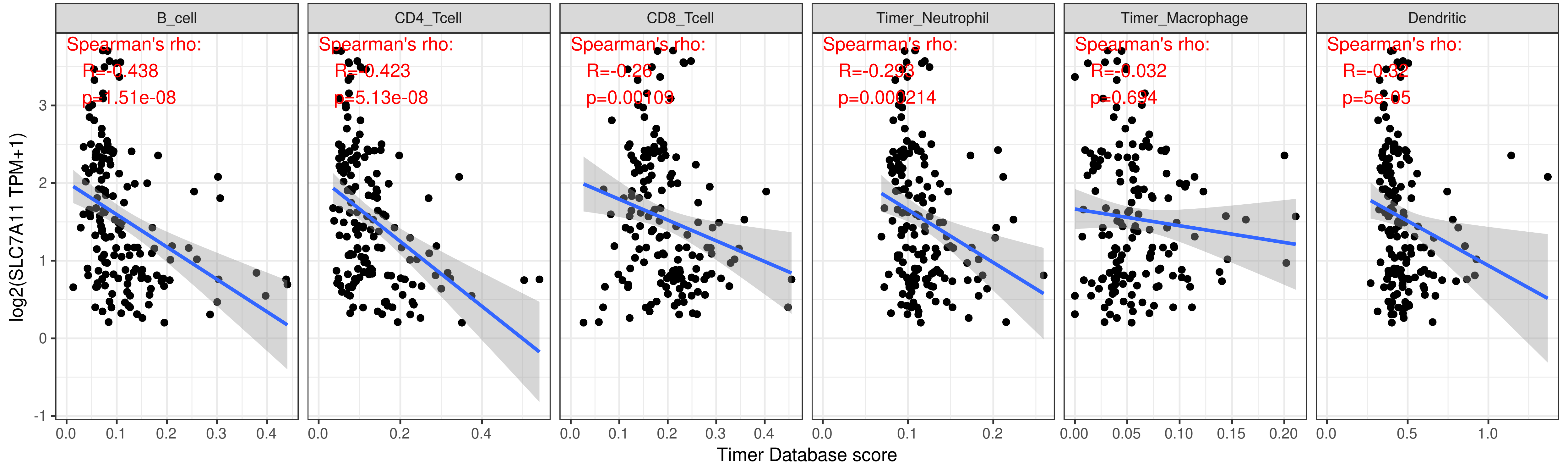
B_cell
Spearman's rho:
R=-0.438
p=1.51e-08
CD4_Tcell
Spearman's rho:
R=-0.423
p=5.13e-08
CD8_Tcell
Spearman's rho:
R=-0.26
p=0.00109
Timer_Neutrophil
Spearman's rho:
R=-0.293
p=0.000214
Timer_Macrophage
Spearman's rho:
R=-0.032
p=0.694
Dendritic
Spearman's rho:
R=-0.32
p=5e-05
log2(SLC7A11 TPM+1)
Timer Database score

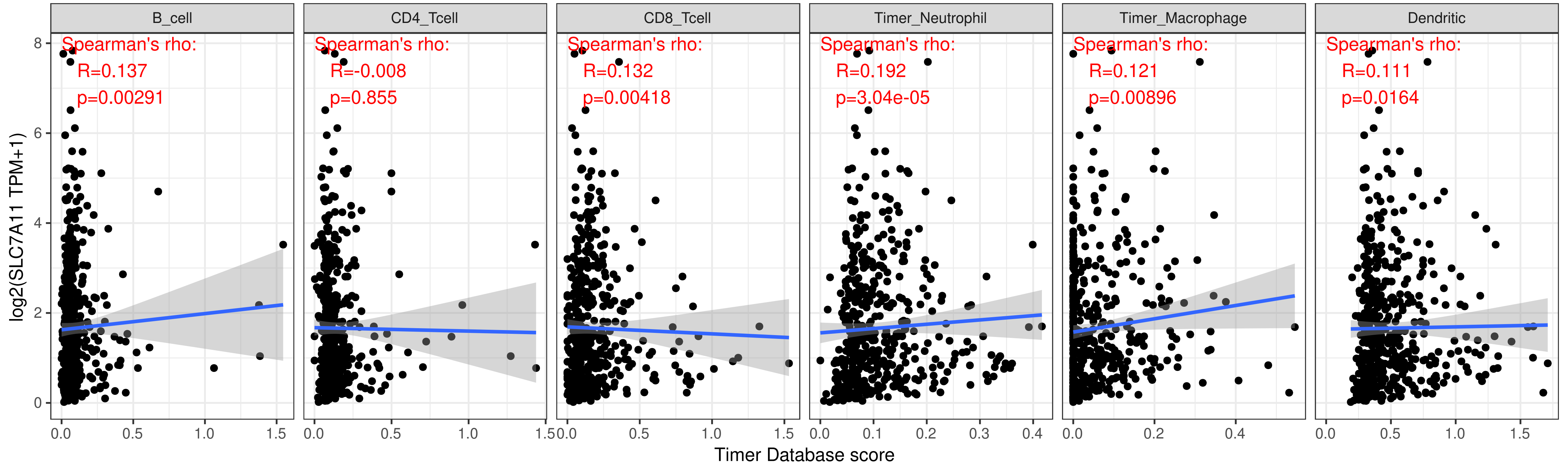
B_cell
CD4_Tcell
CD8_Tcell
Timer_Neutrophil
Timer_Macrophage
Dendritic
Spearman's rho:
R=0.137
p=0.00291
Spearman's rho:
R=-0.008
p=0.855
Spearman's rho:
R=0.132
p=0.00418
Spearman's rho:
R=0.192
p=3.04e-05
Spearman's rho:
R=0.121
p=0.00896
Spearman's rho:
R=0.111
p=0.0164
log2(SLC7A11 TPM+1)
Timer Database score

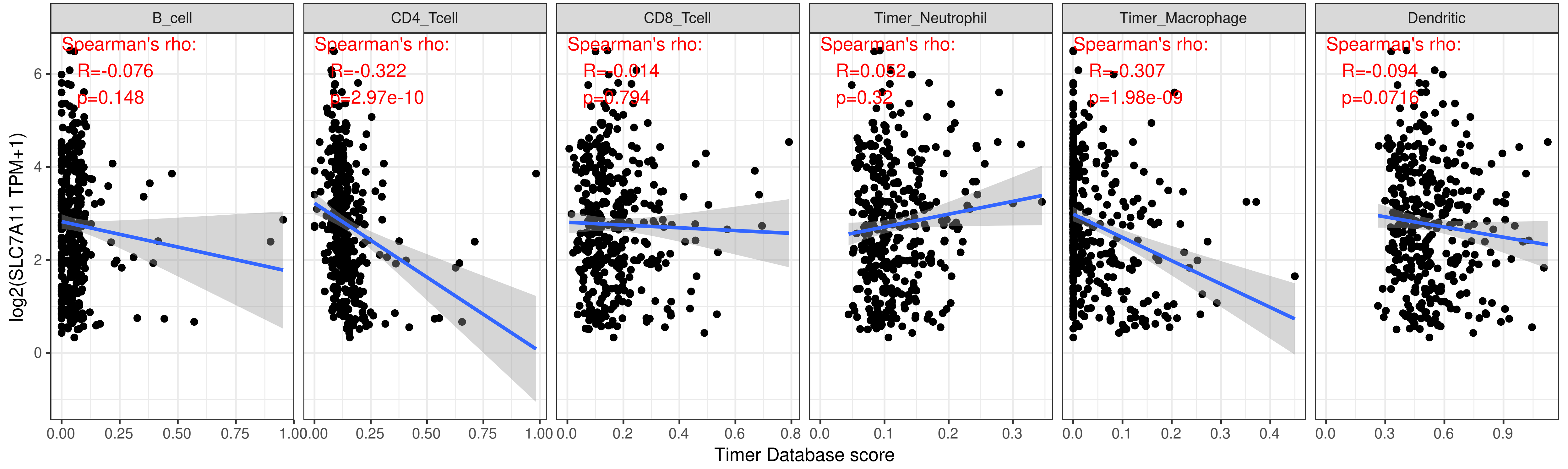
B_cell
CD4_Tcell
CD8_Tcell
Timer_Neutrophil
Timer_Macrophage
Dendritic
Spearman's rho:
R=-0.076
p=0.148
Spearman's rho:
R=-0.322
p=2.97e-10
Spearman's rho:
R=-0.014
p=0.794
Spearman's rho:
R=0.052
p=0.32
Spearman's rho:
R=-0.307
p=1.98e-09
Spearman's rho:
R=-0.094
p=0.0716
log2(SLC7A11 TPM+1)
Timer Database score

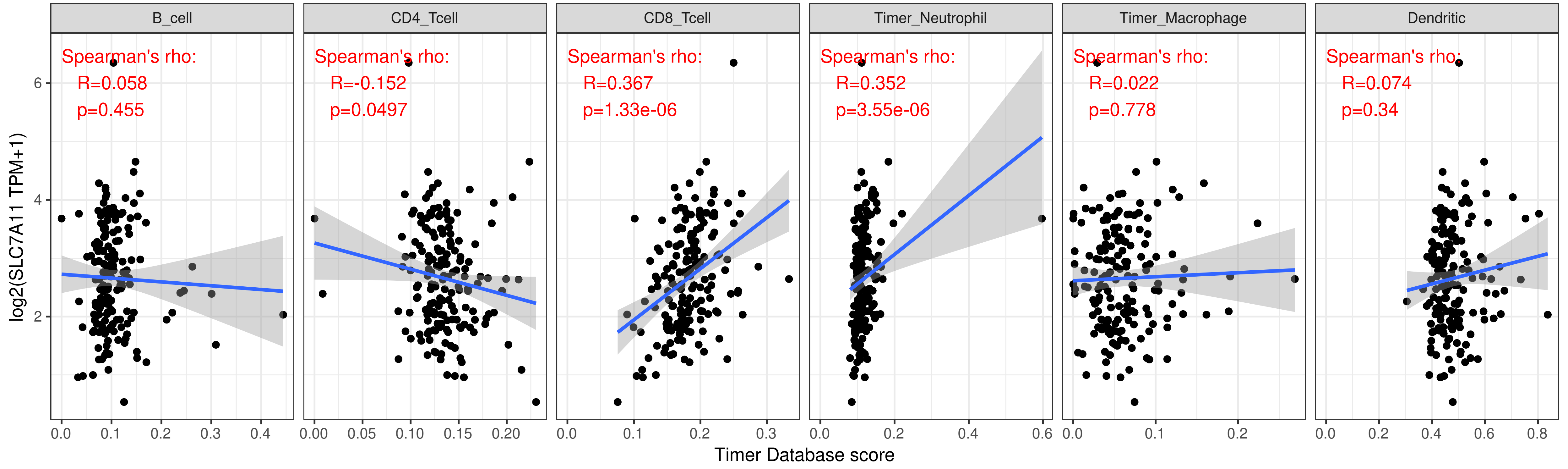
B_cell
CD4_Tcell
CD8_Tcell
Timer_Neutrophil
Timer_Macrophage
Dendritic
Spearman's rho:
R=0.058
p=0.455
Spearman's rho:
R=-0.152
p=0.0497
Spearman's rho:
R=0.367
p=1.33e-06
Spearman's rho:
R=0.352
p=3.55e-06
Spearman's rho:
R=0.022
p=0.778
Spearman's rho:
R=0.074
p=0.34
log2(SLC7A11 TPM+1)
Timer Database score

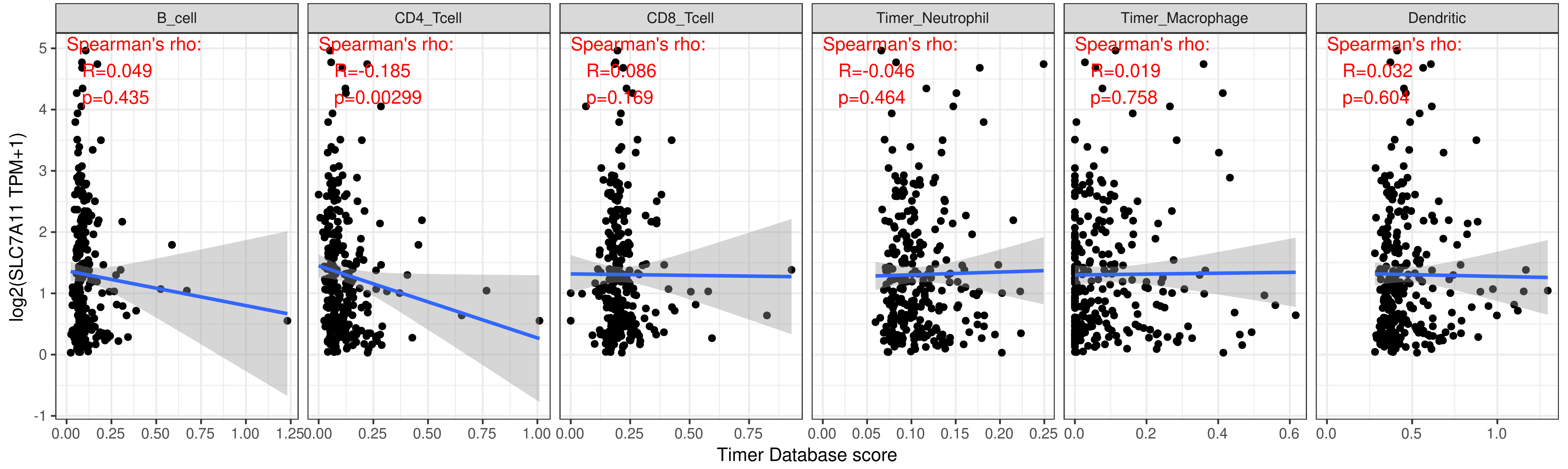

B_cell
CD4_Tcell
CD8_Tcell
Timer_Neutrophil
Timer_Macrophage
Dendritic
Spearman's rho:
R=0.049
p=0.435
Spearman's rho:
R=-0.185
p=0.00299
Spearman's rho:
R=0.086
p=0.169
Spearman's rho:
R=-0.046
p=0.464
Spearman's rho:
R=0.019
p=0.758
Spearman's rho:
R=0.032
p=0.604
log2(SLC7A11 TPM+1)
Timer Database score

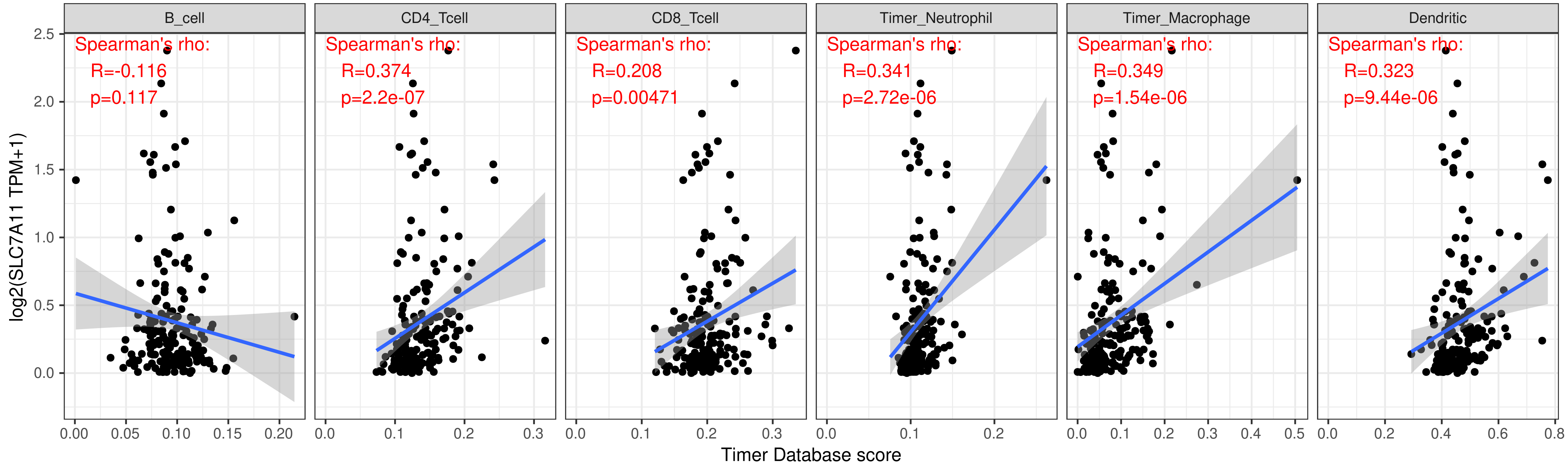
B_cell
CD4_Tcell
CD8_Tcell
Timer_Neutrophil
Timer_Macrophage
Dendritic
Spearman's rho:
R=-0.116
p=0.117
Spearman's rho:
R=0.374
p=2.2e-07
Spearman's rho:
R=0.208
p=0.00471
Spearman's rho:
R=0.341
p=2.72e-06
Spearman's rho:
R=0.349
p=1.54e-06
Spearman's rho:
R=0.323
p=9.44e-06
log2(SLC7A11 TPM+1)
Timer Database score

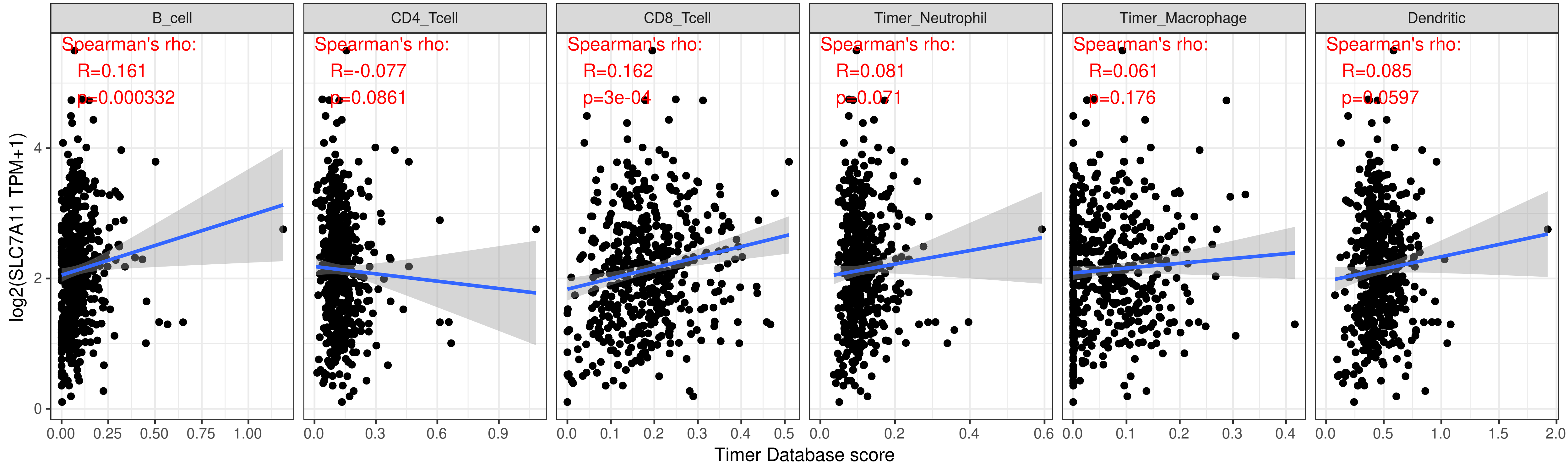

B_cell
Spearman's rho:
R=0.161
p=0.000332
CD4_Tcell
Spearman's rho:
R=-0.077
p=0.0861
CD8_Tcell
Spearman's rho:
R=0.162
p=3e-04
Timer_Neutrophil
Spearman's rho:
R=0.081
p=0.071
Timer_Macrophage
Spearman's rho:
R=0.061
p=0.176
Dendritic
Spearman's rho:
R=0.085
p=0.0597
log2(SLC7A11 TPM+1)
Timer Database score

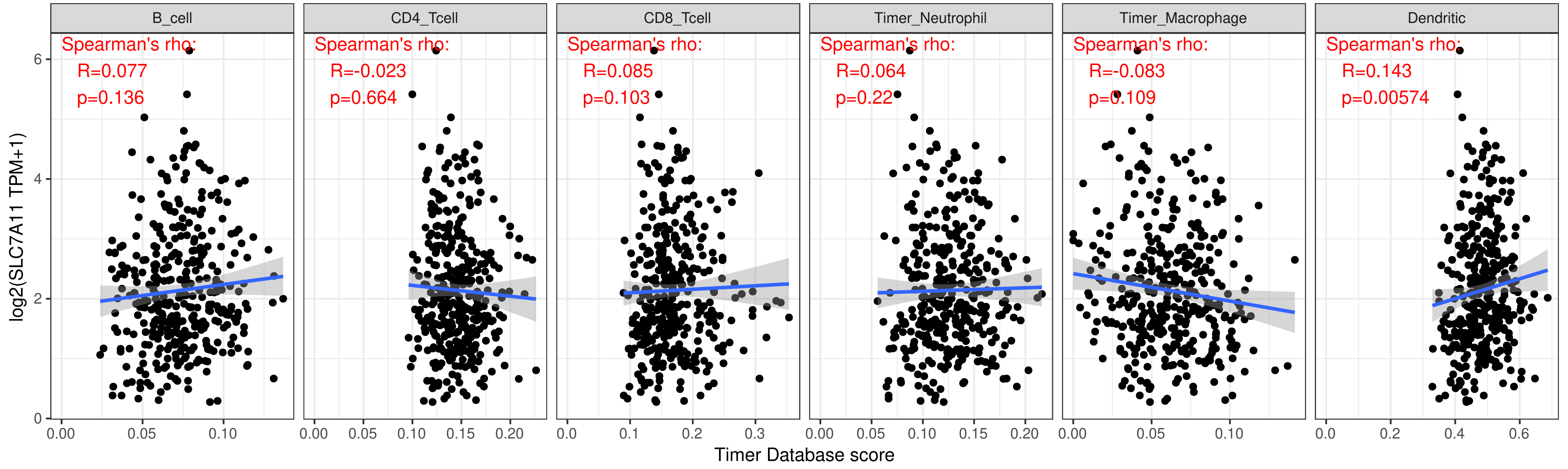
B_cell
CD4_Tcell
CD8_Tcell
Timer_Neutrophil
Timer_Macrophage
Dendritic
Spearman's rho:
R=0.077
p=0.136
Spearman's rho:
R=-0.023
p=0.664
Spearman's rho:
R=0.085
p=0.103
Spearman's rho:
R=0.064
p=0.22
Spearman's rho:
R=-0.083
p=0.109
Spearman's rho:
R=0.143
p=0.00574
log2(SLC7A11 TPM+1)
Timer Database score

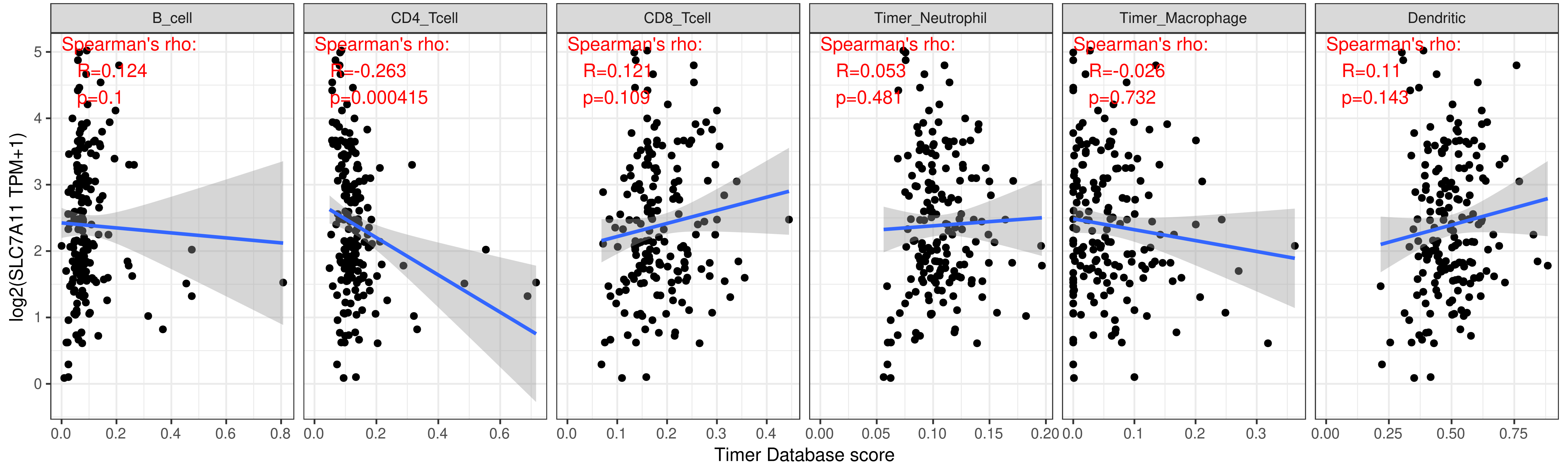

B_cell
Spearman's rho:
R=0.124
p=0.1
CD4_Tcell
Spearman's rho:
R=-0.263
p=0.000415
CD8_Tcell
Spearman's rho:
R=0.121
p=0.109
Timer_Neutrophil
Spearman's rho:
R=0.053
p=0.481
Timer_Macrophage
Spearman's rho:
R=-0.026
p=0.732
Dendritic
Spearman's rho:
R=0.11
p=0.143
log2(SLC7A11 TPM+1)
Timer Database score

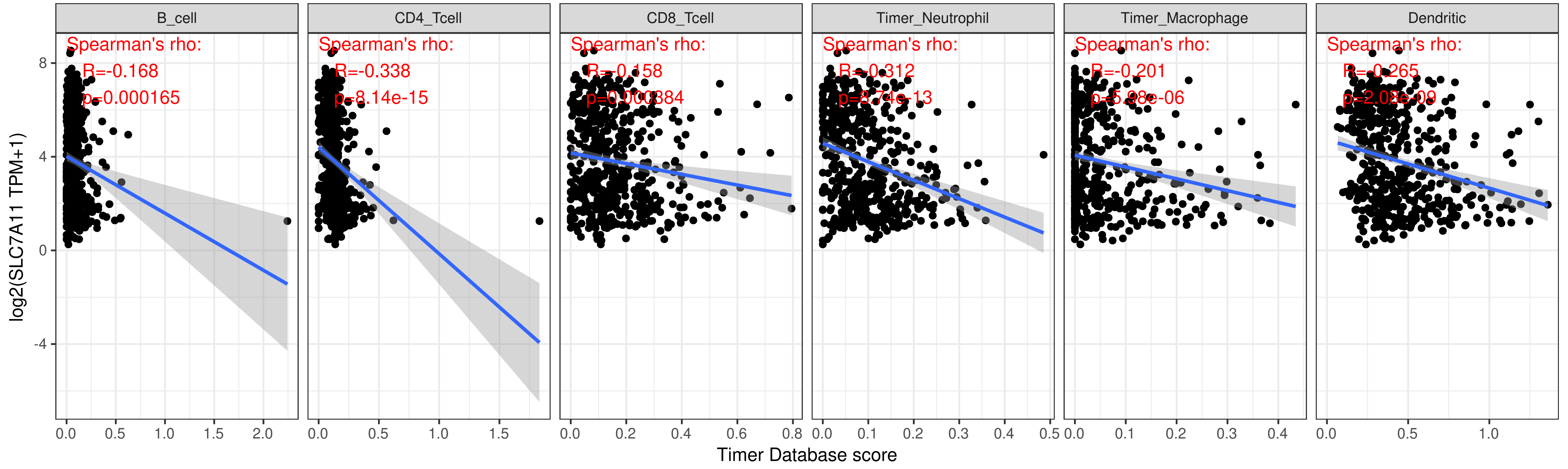

B_cell
CD4_Tcell
CD8_Tcell
Timer_Neutrophil
Timer_Macrophage
Dendritic
Spearman's rho:
R=-0.168
p=0.000165
Spearman's rho:
R=-0.338
p=8.14e-15
Spearman's rho:
R=-0.158
p=0.000384
Spearman's rho:
R=-0.312
p=8.74e-13
Spearman's rho:
R=-0.201
p=5.98e-06
Spearman's rho:
R=-0.265
p=2.08e-09
log2(SLC7A11 TPM+1)
Timer Database score

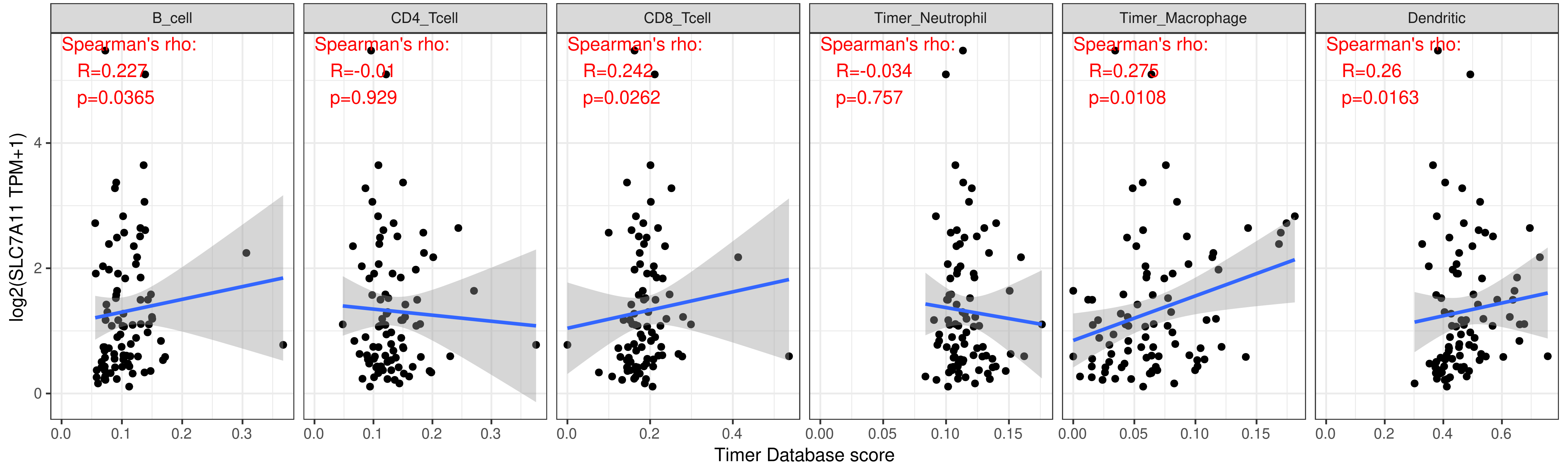
B_cell
Spearman's rho:
R=0.227
p=0.0365
CD4_Tcell
Spearman's rho:
R=-0.01
p=0.929
CD8_Tcell
Spearman's rho:
R=0.242
p=0.0262
Timer_Neutrophil
Spearman's rho:
R=-0.034
p=0.757
Timer_Macrophage
Spearman's rho:
R=0.275
p=0.0108
Dendritic
Spearman's rho:
R=0.26
p=0.0163
log2(SLC7A11 TPM+1)
Timer Database score

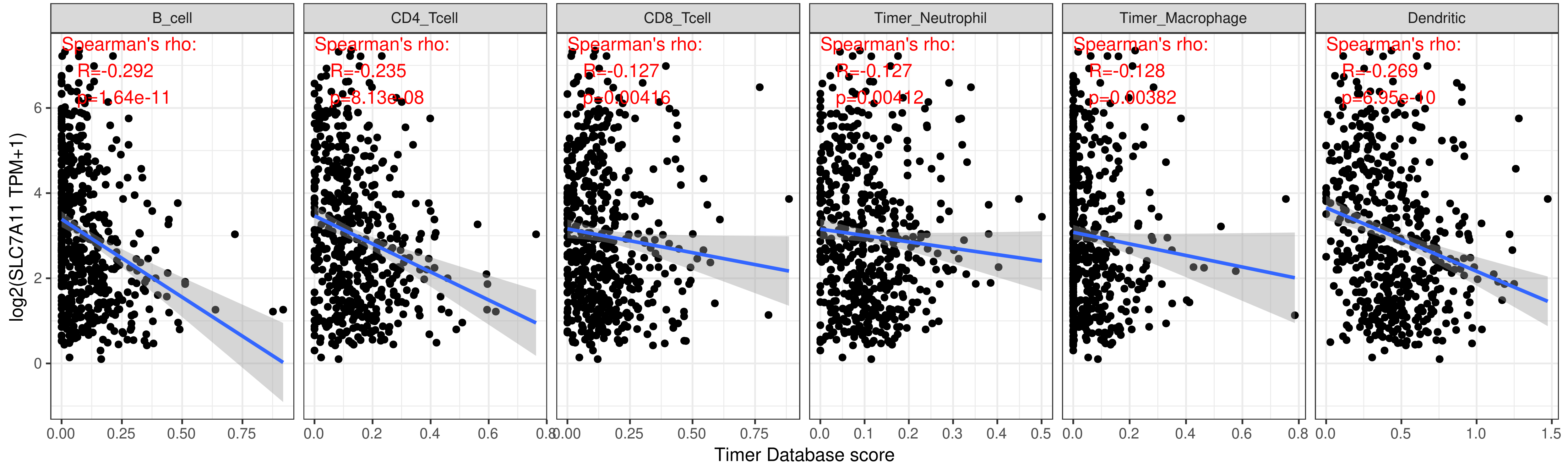

B_cell
CD4_Tcell
CD8_Tcell
Timer_Neutrophil
Timer_Macrophage
Dendritic
Spearman's rho:
R=-0.292
p=1.64e-11
Spearman's rho:
R=-0.235
p=8.13e-08
Spearman's rho:
R=-0.127
p=0.00416
Spearman's rho:
R=-0.127
p=0.00412
Spearman's rho:
R=-0.128
p=0.00382
Spearman's rho:
R=-0.269
p=6.95e-10
log2(SLC7A11 TPM+1)
Timer Database score

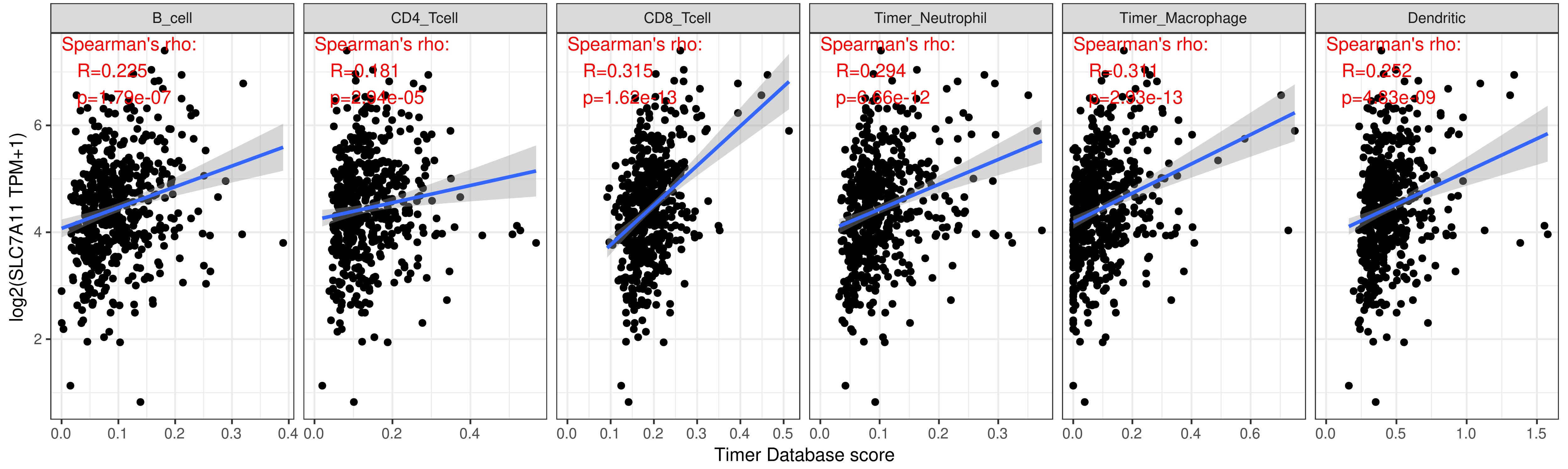
B_cell
CD4_Tcell
CD8_Tcell
Timer_Neutrophil
Timer_Macrophage
Dendritic
Spearman's rho:
R=0.225
p=1.79e-07
Spearman's rho:
R=0.181
p=2.94e-05
Spearman's rho:
R=0.315
p=1.62e-13
Spearman's rho:
R=0.294
p=6.66e-12
Spearman's rho:
R=0.311
p=2.93e-13
Spearman's rho:
R=0.252
p=4.83e-09
log2(SLC7A11 TPM+1)
Timer Database score

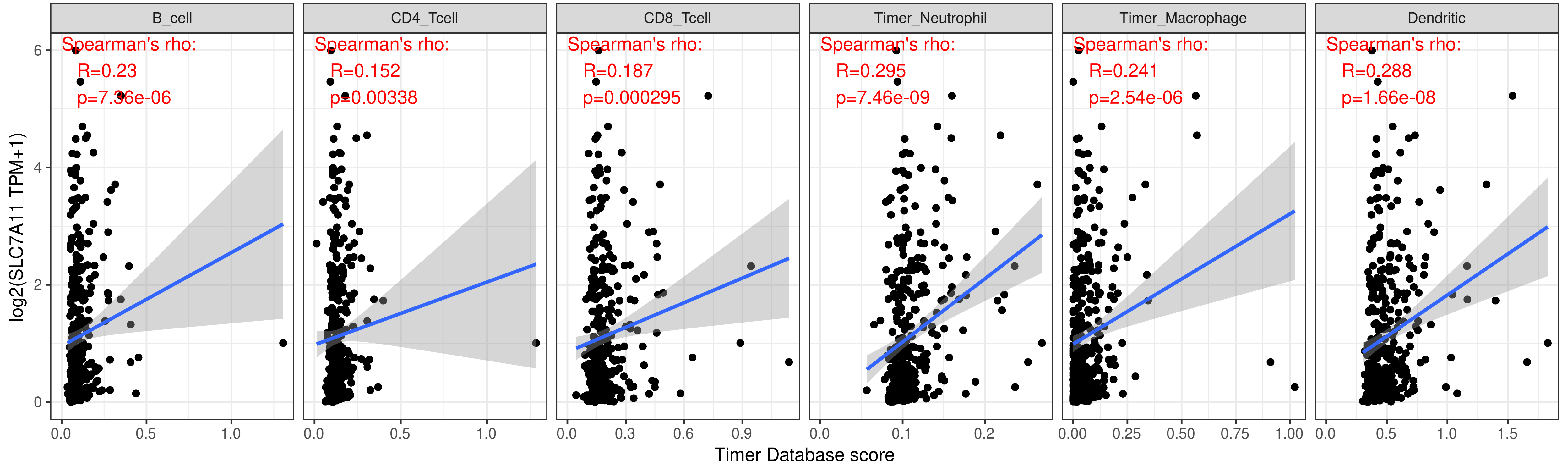

B_cell
CD4_Tcell
CD8_Tcell
Timer_Neutrophil
Timer_Macrophage
Dendritic
Spearman's rho:
R=0.23
p=7.36e-06
Spearman's rho:
R=0.152
p=0.00338
Spearman's rho:
R=0.187
p=0.000295
Spearman's rho:
R=0.295
p=7.46e-09
Spearman's rho:
R=0.241
p=2.54e-06
Spearman's rho:
R=0.288
p=1.66e-08
log2(SLC7A11 TPM+1)
Timer Database score

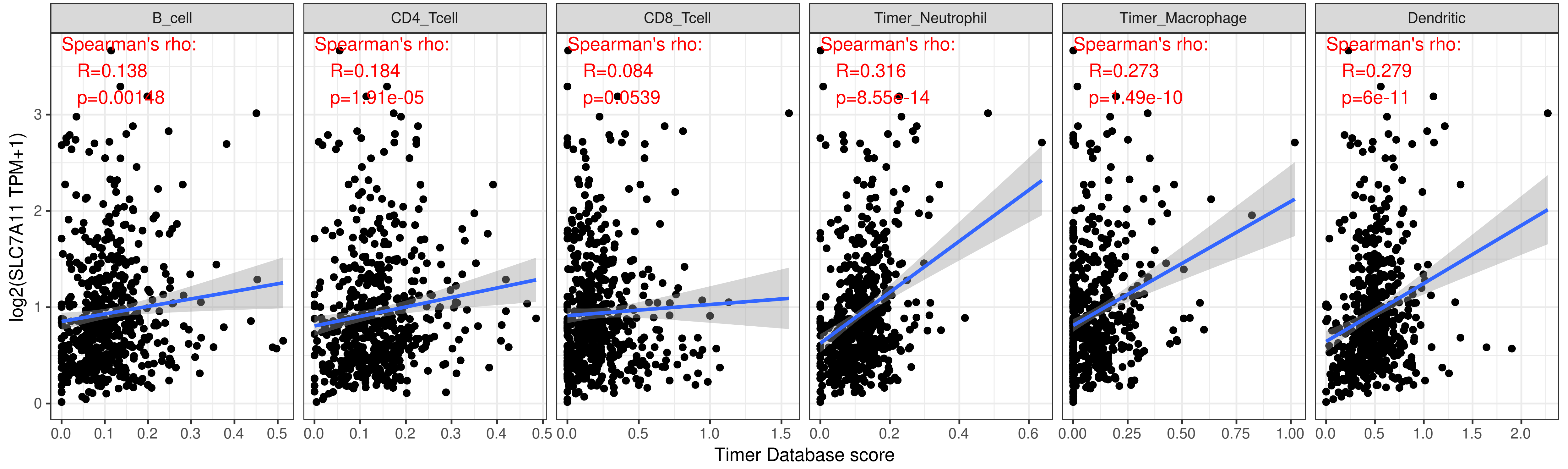
B_cell
CD4_Tcell
CD8_Tcell
Timer_Neutrophil
Timer_Macrophage
Dendritic
Spearman's rho:
R=0.138
p=0.00148
Spearman's rho:
R=0.184
p=1.91e-05
Spearman's rho:
R=0.084
p=0.0539
Spearman's rho:
R=0.316
p=8.55e-14
Spearman's rho:
R=0.273
p=1.49e-10
Spearman's rho:
R=0.279
p=6e-11
log2(SLC7A11 TPM+1)
Timer Database score

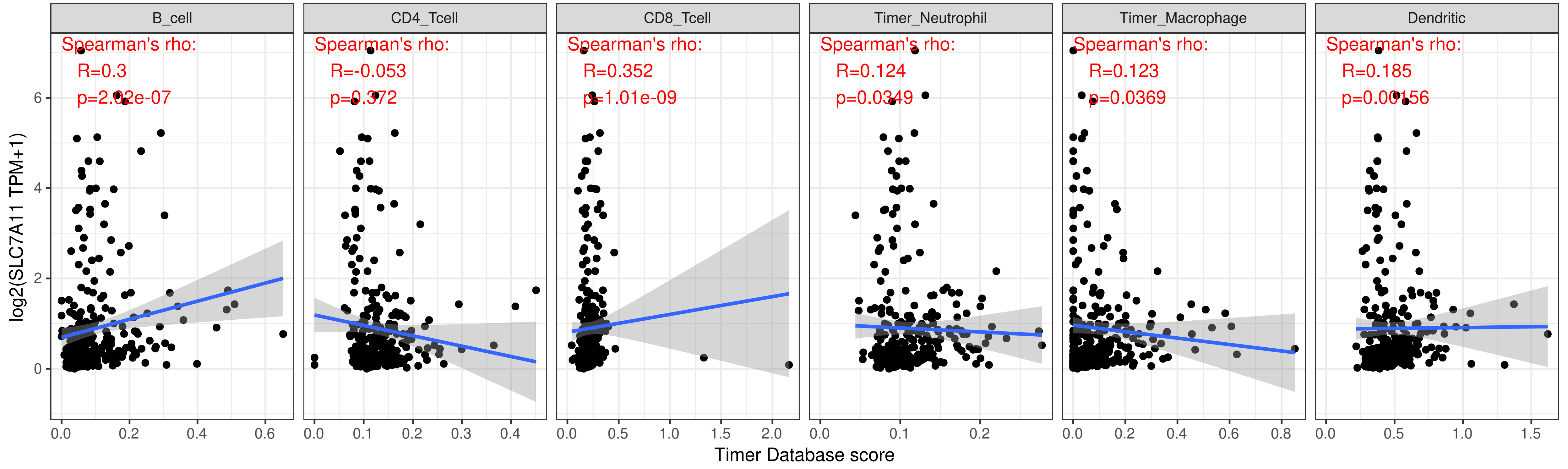

B_cell
CD4_Tcell
CD8_Tcell
Timer_Neutrophil
Timer_Macrophage
Dendritic
Spearman's rho:
R=0.3
p=2.02e-07
Spearman's rho:
R=-0.053
p=0.372
Spearman's rho:
R=0.352
p=1.01e-09
Spearman's rho:
R=0.124
p=0.0349
Spearman's rho:
R=0.123
p=0.0369
Spearman's rho:
R=0.185
p=0.00156
log2(SLC7A11 TPM+1)
Timer Database score

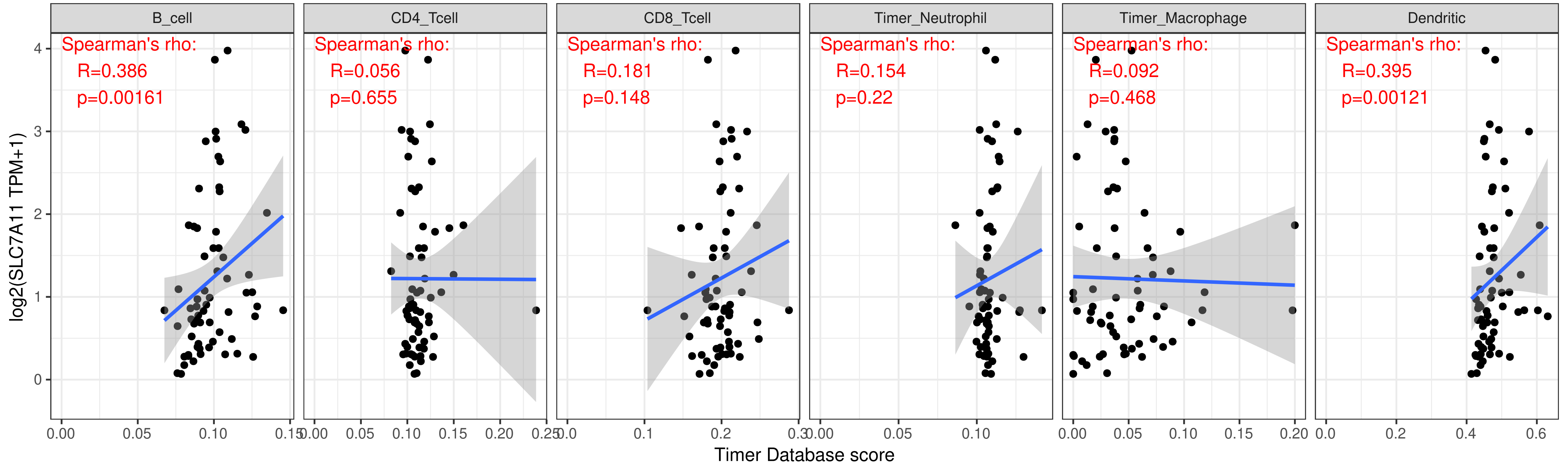
B_cell
CD4_Tcell
CD8_Tcell
Timer_Neutrophil
Timer_Macrophage
Dendritic
Spearman's rho:
R=0.386
p=0.00161
Spearman's rho:
R=0.056
p=0.655
Spearman's rho:
R=0.181
p=0.148
Spearman's rho:
R=0.154
p=0.22
Spearman's rho:
R=0.092
p=0.468
Spearman's rho:
R=0.395
p=0.00121
log2(SLC7A11 TPM+1)
Timer Database score

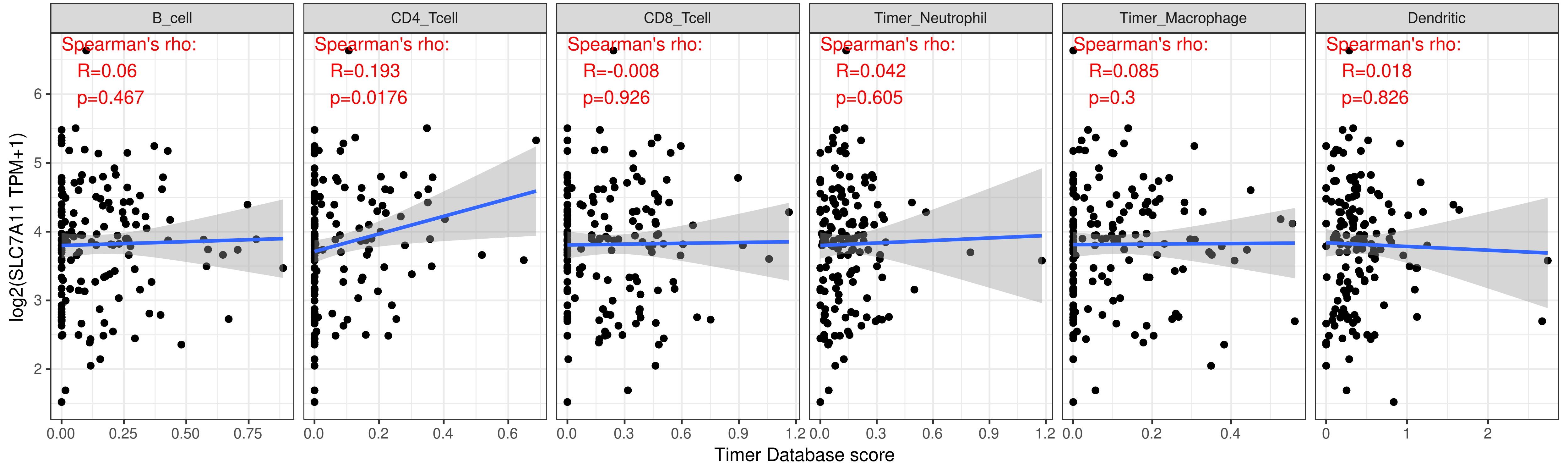
B_cell
CD4_Tcell
CD8_Tcell
Timer_Neutrophil
Timer_Macrophage
Dendritic
Spearman's rho:
R=0.06
p=0.467
Spearman's rho:
R=0.193
p=0.0176
Spearman's rho:
R=-0.008
p=0.926
Spearman's rho:
R=0.042
p=0.605
Spearman's rho:
R=0.085
p=0.3
Spearman's rho:
R=0.018
p=0.826
log2(SLC7A11 TPM+1)
Timer Database score

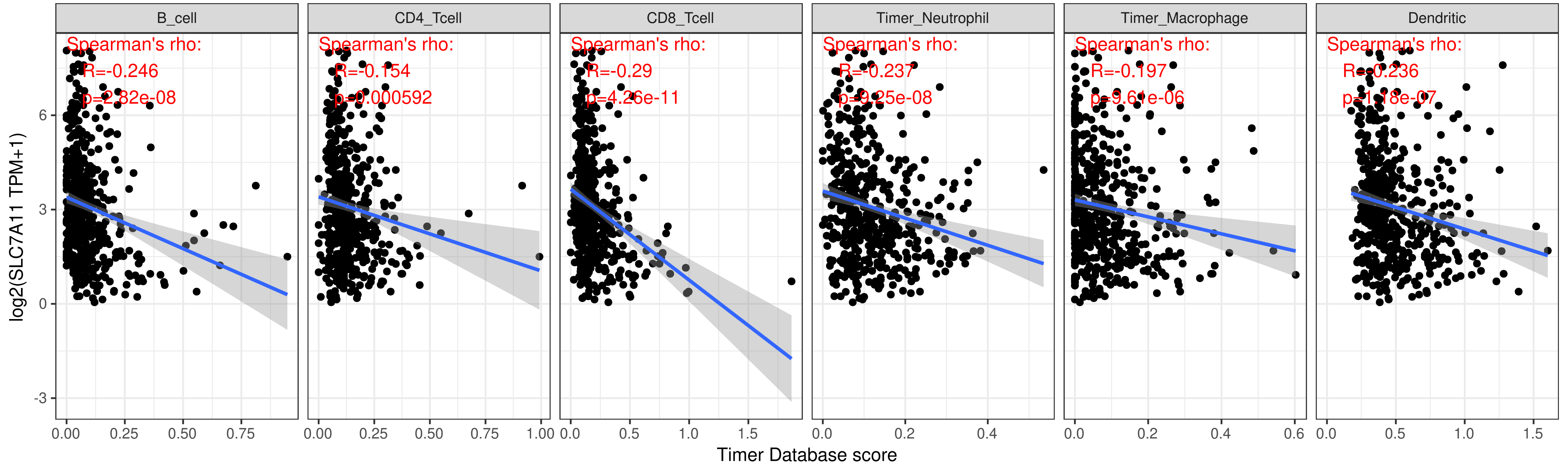

B_cell
CD4_Tcell
CD8_Tcell
Timer_Neutrophil
Timer_Macrophage
Dendritic
Spearman's rho:
R=-0.246
p=2.82e-08
Spearman's rho:
R=-0.154
p=0.000592
Spearman's rho:
R=-0.29
p=4.26e-11
Spearman's rho:
R=-0.237
p=9.25e-08
Spearman's rho:
R=-0.197
p=9.61e-06
Spearman's rho:
R=-0.236
p=1.18e-07
log2(SLC7A11 TPM+1)
Timer Database score

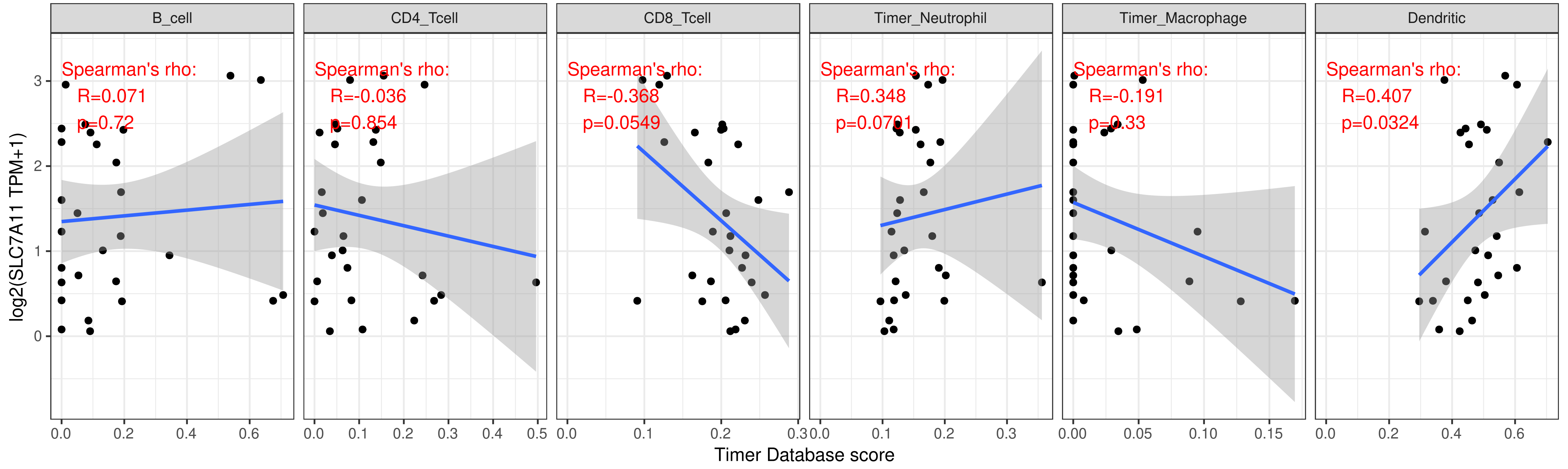
B_cell
CD4_Tcell
CD8_Tcell
Timer_Neutrophil
Timer_Macrophage
Dendritic
Spearman's rho:
R=0.071
p=0.72
Spearman's rho:
R=-0.036
p=0.854
Spearman's rho:
R=-0.368
p=0.0549
Spearman's rho:
R=0.348
p=0.0701
Spearman's rho:
R=-0.191
p=0.33
Spearman's rho:
R=0.407
p=0.0324
log2(SLC7A11 TPM+1)
Timer Database score

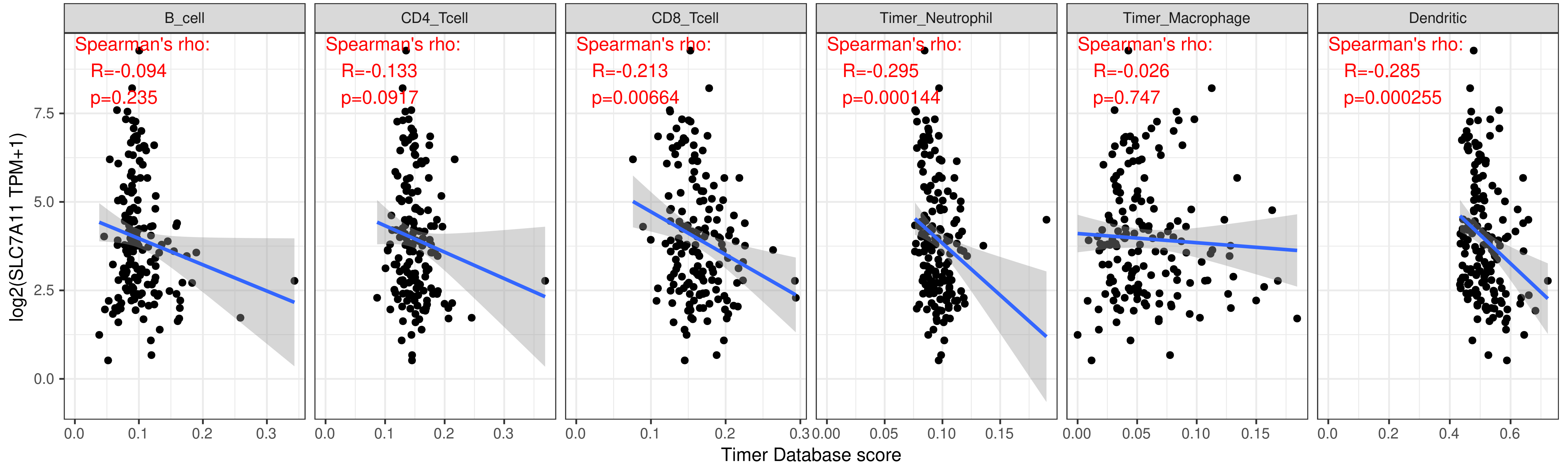
B_cell
CD4_Tcell
CD8_Tcell
Timer_Neutrophil
Timer_Macrophage
Dendritic
Spearman's rho:
R=-0.094
p=0.235
Spearman's rho:
R=-0.133
p=0.0917
Spearman's rho:
R=-0.213
p=0.00664
Spearman's rho:
R=-0.295
p=0.000144
Spearman's rho:
R=-0.026
p=0.747
Spearman's rho:
R=-0.285
p=0.000255
log2(SLC7A11 TPM+1)
Timer Database score

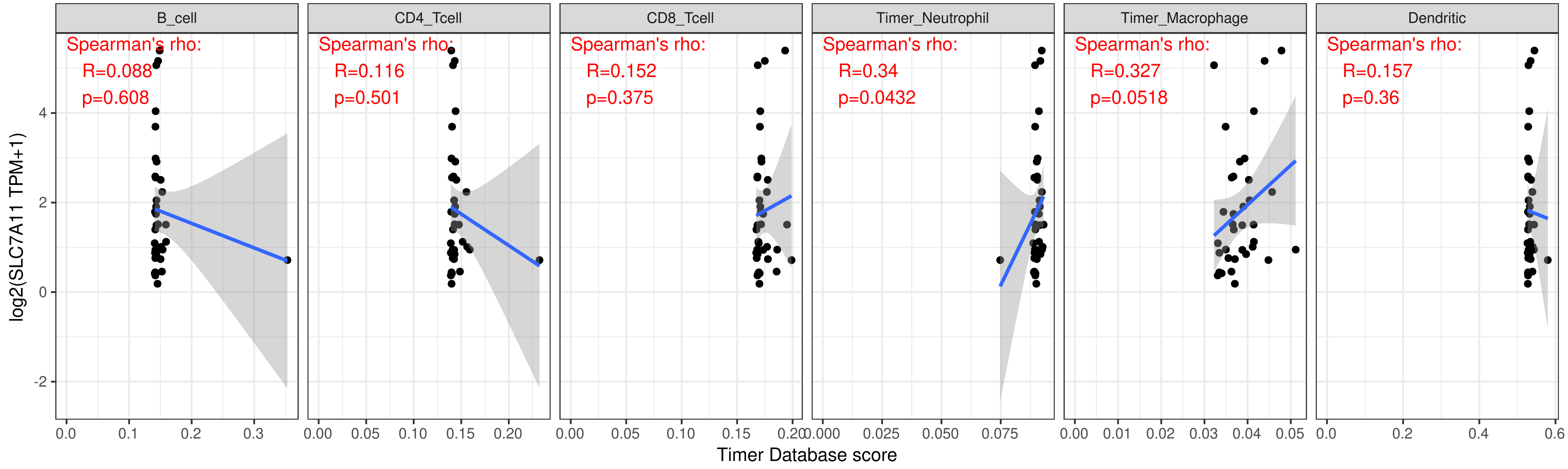
B_cell
Spearman's rho:
R=0.088
p=0.608
CD4_Tcell
Spearman's rho:
R=0.116
p=0.501
CD8_Tcell
Spearman's rho:
R=0.152
p=0.375
Timer_Neutrophil
Spearman's rho:
R=0.34
p=0.0432
Timer_Macrophage
Spearman's rho:
R=0.327
p=0.0518
Dendritic
Spearman's rho:
R=0.157
p=0.36
log2(SLC7A11 TPM+1)
Timer Database score

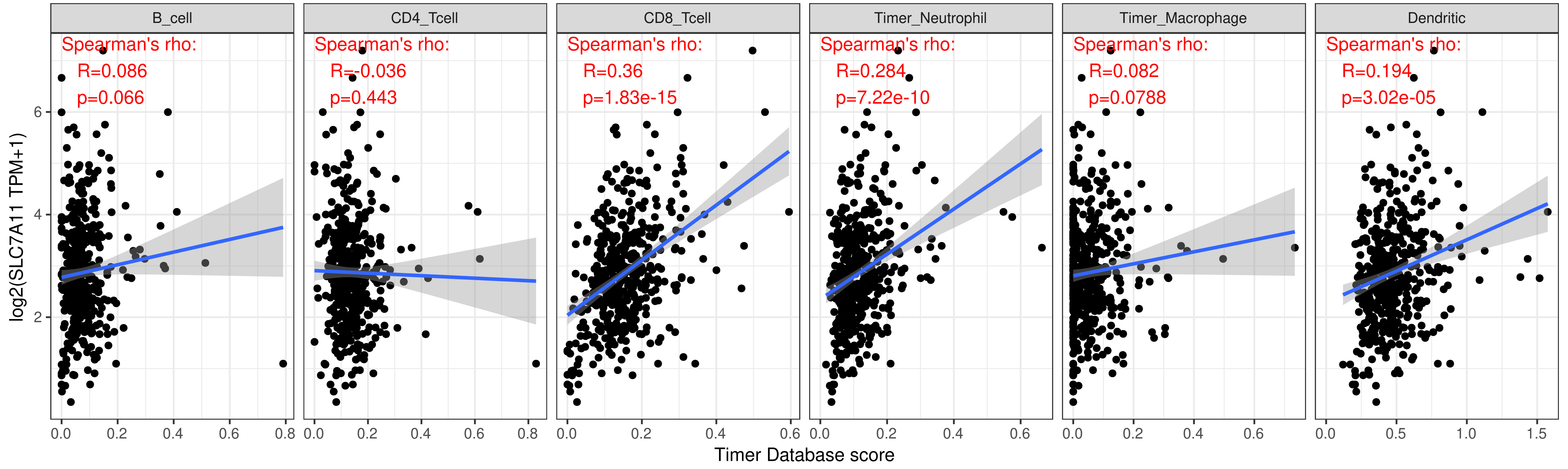
B_cell
CD4_Tcell
CD8_Tcell
Timer_Neutrophil
Timer_Macrophage
Dendritic
Spearman's rho:
R=0.086
p=0.066
Spearman's rho:
R=-0.036
p=0.443
Spearman's rho:
R=0.36
p=1.83e-15
Spearman's rho:
R=0.284
p=7.22e-10
Spearman's rho:
R=0.082
p=0.0788
Spearman's rho:
R=0.194
p=3.02e-05
log2(SLC7A11 TPM+1)
Timer Database score

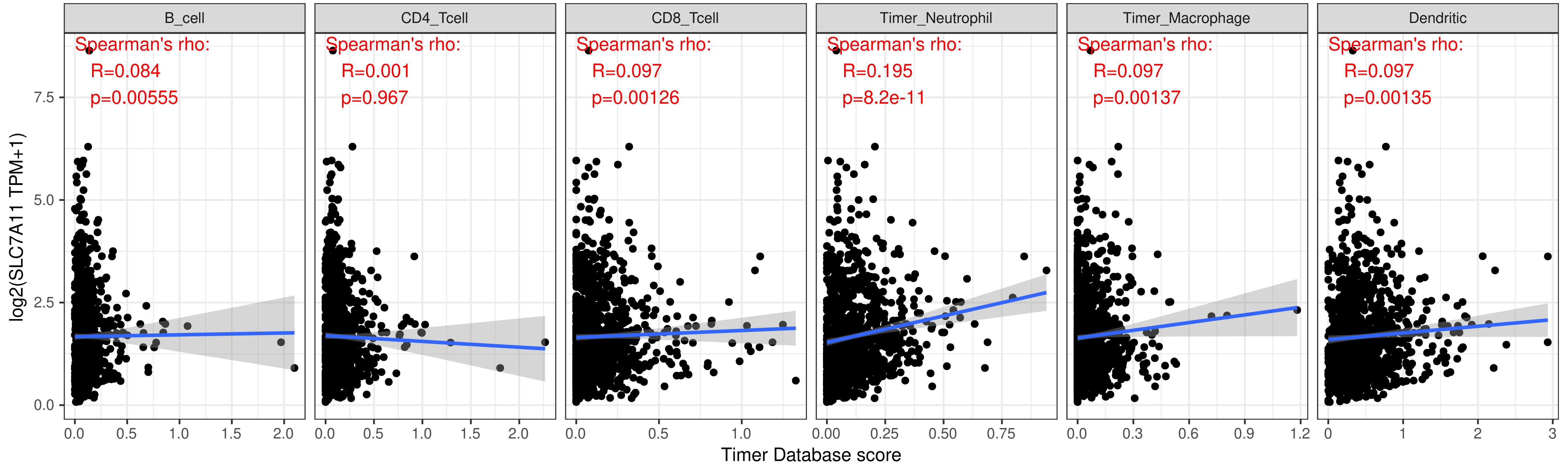
B_cell
CD4_Tcell
CD8_Tcell
Timer_Neutrophil
Timer_Macrophage
Dendritic
Spearman's rho:
R=0.084
p=0.00555
Spearman's rho:
R=0.001
p=0.967
Spearman's rho:
R=0.097
p=0.00126
Spearman's rho:
R=0.195
p=8.2e-11
Spearman's rho:
R=0.097
p=0.00137
Spearman's rho:
R=0.097
p=0.00135
log2(SLC7A11 TPM+1)
Timer Database score

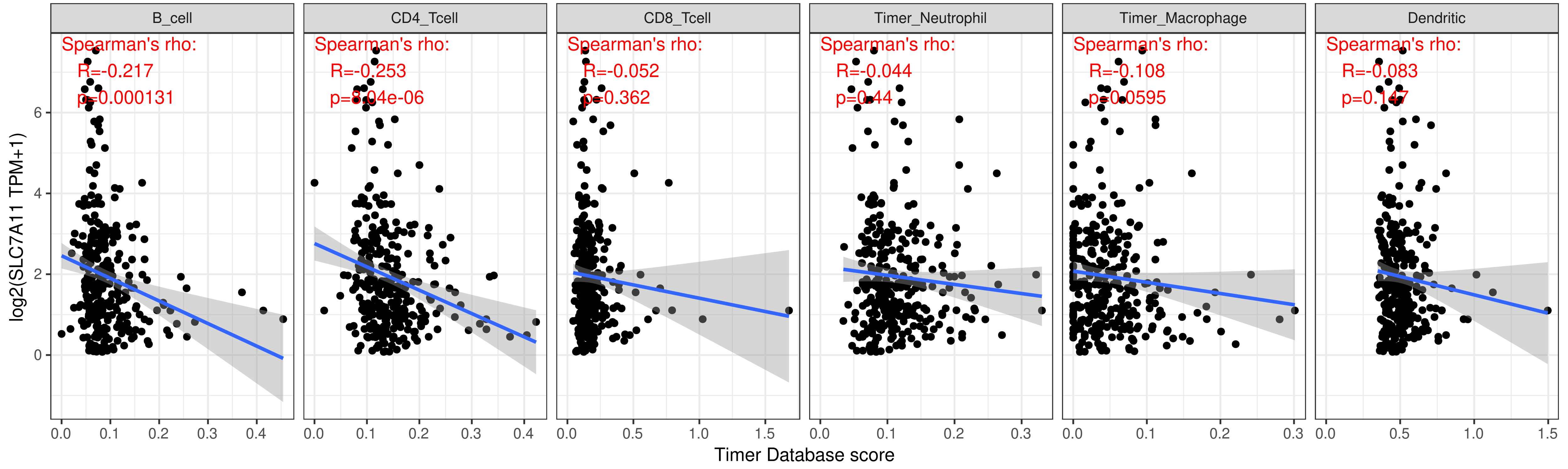
B_cell
CD4_Tcell
CD8_Tcell
Timer_Neutrophil
Timer_Macrophage
Dendritic
Spearman's rho:
R=-0.217
p=0.000131
Spearman's rho:
R=-0.253
p=8.04e-06
Spearman's rho:
R=-0.052
p=0.362
Spearman's rho:
R=-0.044
p=0.44
Spearman's rho:
R=-0.108
p=0.0595
Spearman's rho:
R=-0.083
p=0.147
log2(SLC7A11 TPM+1)
Timer Database score

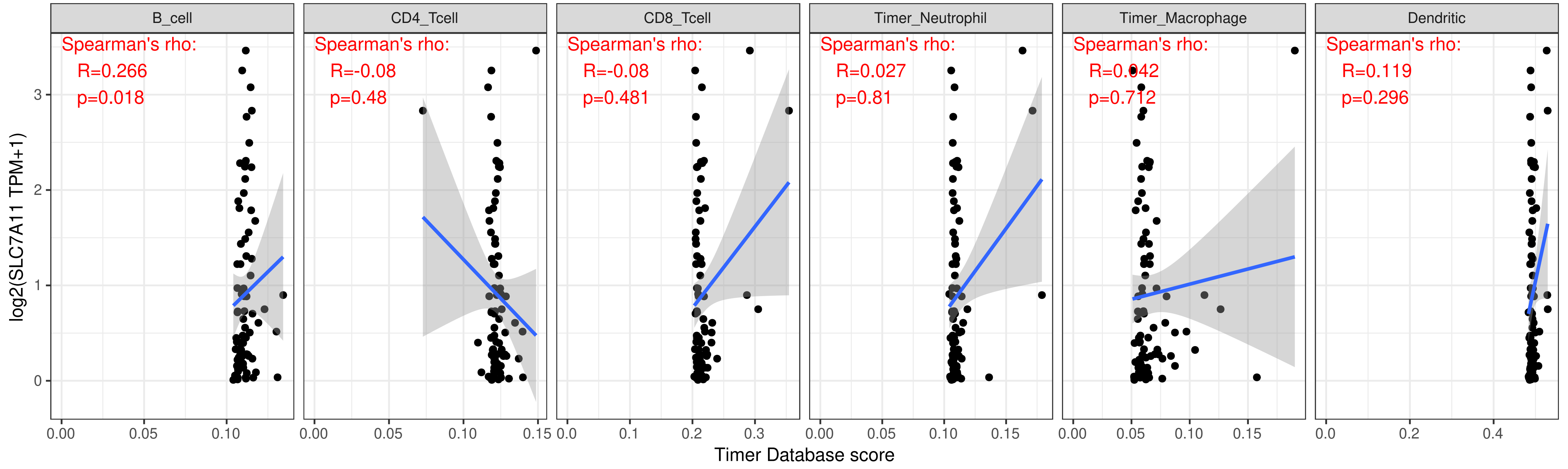
B_cell
CD4_Tcell
CD8_Tcell
Timer_Neutrophil
Timer_Macrophage
Dendritic
Spearman's rho:
R=0.266
p=0.018
Spearman's rho:
R=-0.08
p=0.48
Spearman's rho:
R=-0.08
p=0.481
Spearman's rho:
R=0.027
p=0.81
Spearman's rho:
R=0.042
p=0.712
Spearman's rho:
R=0.119
p=0.296
log2(SLC7A11 TPM+1)
Timer Database score

Supplementary figure 4. The correlation coefficient between SLC7A11 expression and immune/stromal/estimate scores in the top 3 cancers .

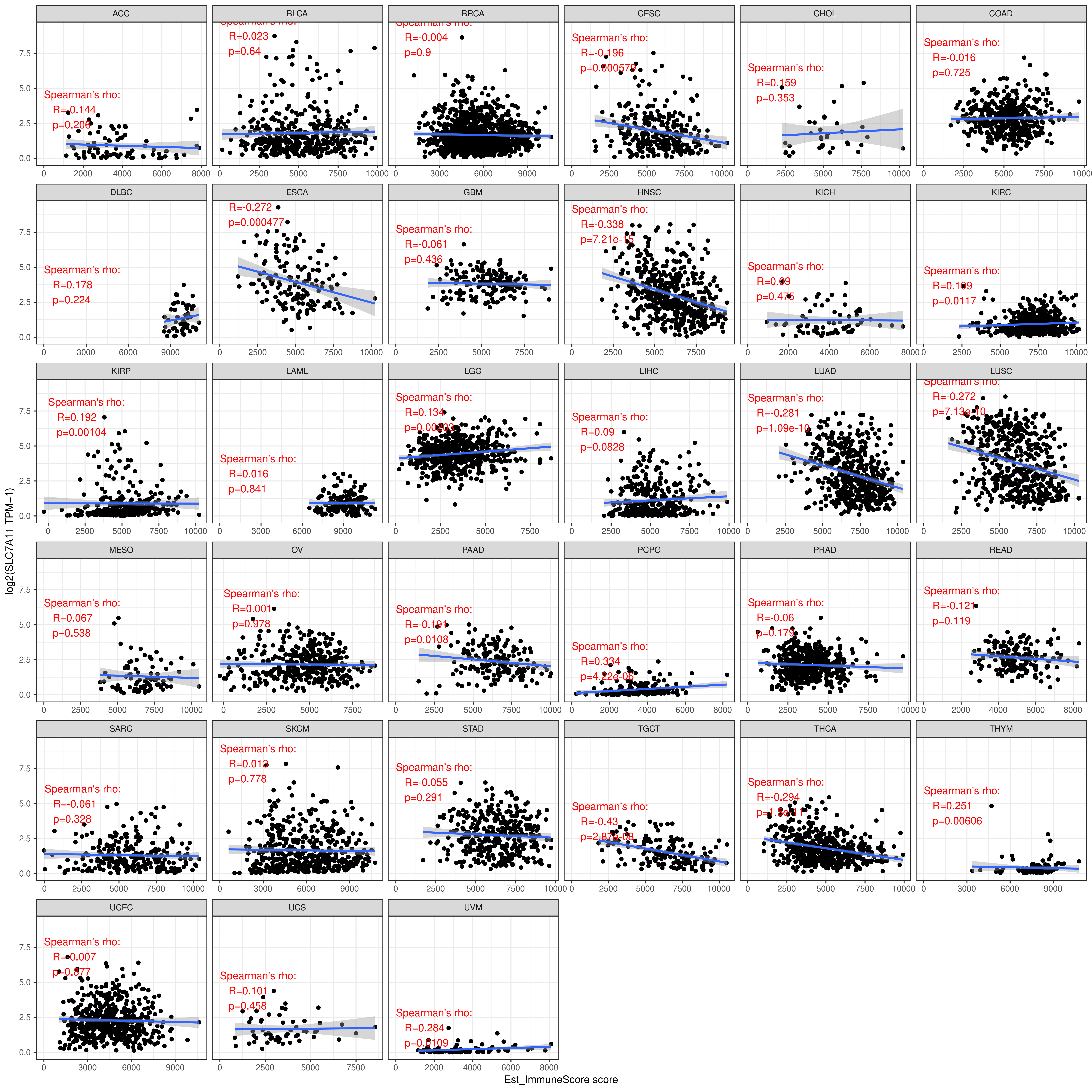

ACC
Spearman's rho:
R=0.144
p=0.206
BLCA
R=0.023
p=0.64
BRCA
R=-0.004
p=0.9
CESC
Spearman's rho:
R=-0.196
p=0.000579
CHOL
Spearman's rho:
R=0.159
p=0.353
COAD
Spearman's rho:
R=-0.016
p=0.725
DLBC
Spearman's rho:
R=0.178
p=0.224
ESCA
R=-0.272
p=0.000477
GBM
Spearman's rho:
R=-0.061
p=0.436
HNSC
Spearman's rho:
R=-0.338
p=7.21e-15
KICH
Spearman's rho:
R=0.09
p=0.475
KIRC
Spearman's rho:
R=0.109
p=0.0117
KIRP
Spearman's rho:
R=0.192
p=0.00104
LAML
Spearman's rho:
R=0.016
p=0.841
LGG
Spearman's rho:
R=0.134
LIHC
Spearman's rho:
R=0.09
p=0.0828
LUAD
Spearman's rho:
R=-0.281
p=1.09e-10
LUSC
R=-0.272
MESO
Spearman's rho:
R=0.067
p=0.538
OV
Spearman's rho:
R=0.001
p=0.978
PAAD
Spearman's rho:
p=0.0108
PCPG
Spearman's rho:
R=0.334
PRAD
Spearman's rho:
R=-0.06
READ
Spearman's rho:
R=-0.121
p=0.119
SARC
Spearman's rho:
R=-0.061
p=0.328
SKCM
Spearman's rho:
p=0.778
STAD
Spearman's rho:
R=-0.055
p=0.291
TGCT
Spearman's rho:
R=-0.43
THCA
Spearman's rho:
R=-0.294
THYM
Spearman's rho:
R=0.251
p=0.00606
UCEC
Spearman's rho:
R=0.007
UCS
Spearman's rho:
R=0.101
p=0.458
UVM
Spearman's rho:
R=0.284
log2(SLC7A11 TPM+1)
Est_ImmuneScore score

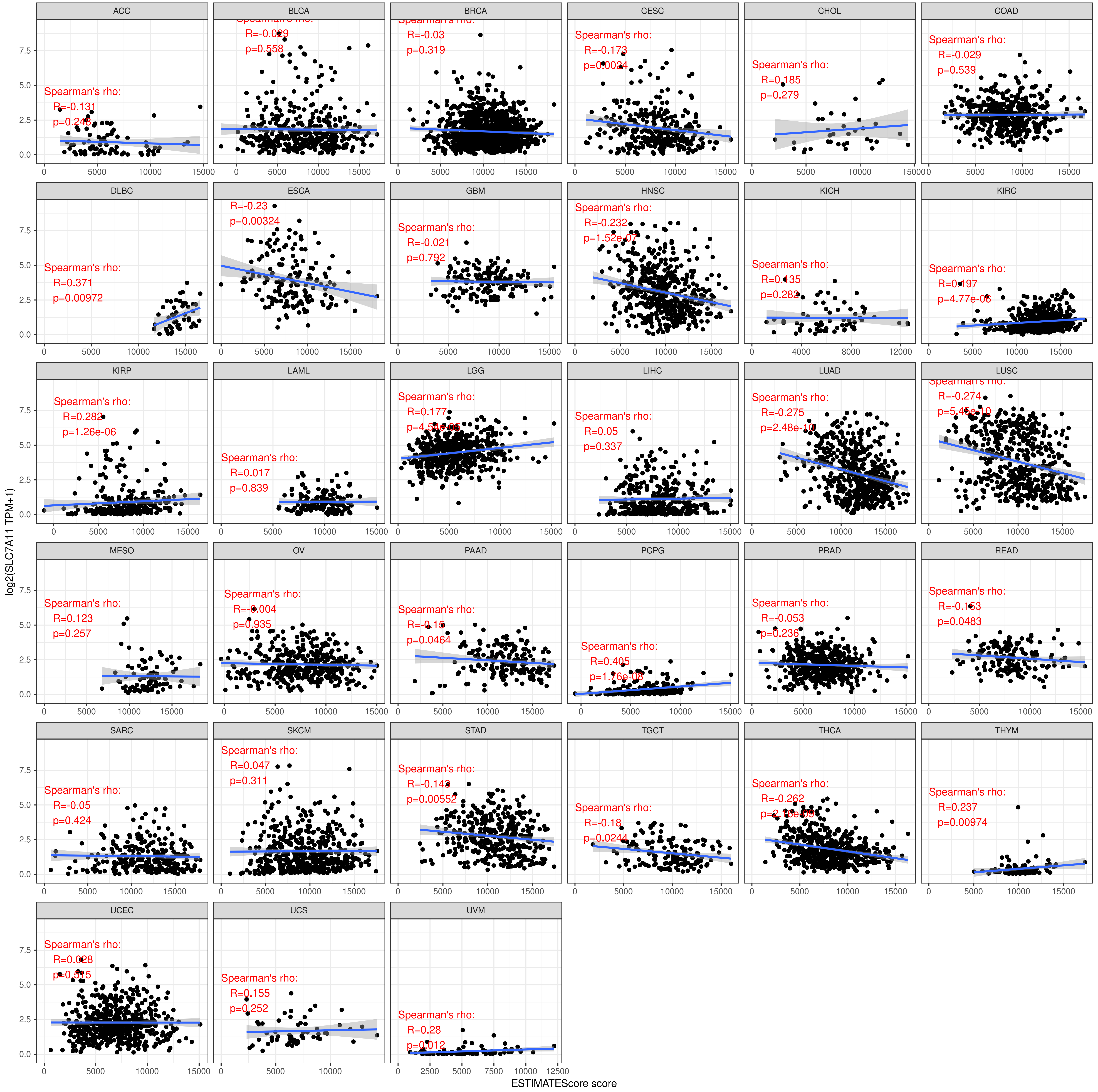
ACC
Spearman's rho:
R=-0.131
p=0.248
BLCA
R=-0.029
p=0.558
BRCA
R=-0.03
p=0.319
CESC
Spearman's rho:
R=-0.173
p=0.0024
CHOL
Spearman's rho:
R=0.185
p=0.279
COAD
Spearman's rho:
R=-0.029
p=0.539
DLBC
Spearman's rho:
R=0.371
p=0.00972
ESCA
R=-0.23
p=0.00324
GBM
Spearman's rho:
R=-0.021
p=0.792
HNSC
Spearman's rho:
R=-0.232
p=1.52e-07
KICH
Spearman's rho:
R=0.135
p=0.282
KIRC
Spearman's rho:
R=0.197
p=4.77e-06
KIRP
Spearman's rho:
R=0.282
p=1.26e-06
LAML
Spearman's rho:
R=0.017
p=0.839
LGG
Spearman's rho:
R=0.177
p=4.51e-05
LIHC
Spearman's rho:
R=0.05
p=0.337
LUAD
Spearman's rho:
R=-0.275
p=2.48e-10
LUSC
R=-0.274
p=5.45e-10
MESO
Spearman's rho:
R=0.123
p=0.257
OV
Spearman's rho:
R=-0.004
p=0.935
PAAD
Spearman's rho:
R=-0.15
p=0.0464
PCPG
Spearman's rho:
R=0.405
p=1.76e-08
PRAD
Spearman's rho:
R=-0.053
p=0.236
READ
Spearman's rho:
R=-0.153
p=0.0483
SARC
Spearman's rho:
R=-0.05
p=0.424
SKCM
Spearman's rho:
R=0.047
p=0.311
STAD
Spearman's rho:
R=-0.143
p=0.00552
TGCT
Spearman's rho:
R=-0.18
p=0.0244
THCA
Spearman's rho:
R=-0.262
THYM
Spearman's rho:
R=0.237
p=0.00974
UCEC
Spearman's rho:
R=0.028
p=0.515
UCS
Spearman's rho:
R=0.155
p=0.252
UVM
Spearman's rho:
R=0.28
p=0.012
log2(SLC7A11 TPM+1)
ESTIMATEScore score

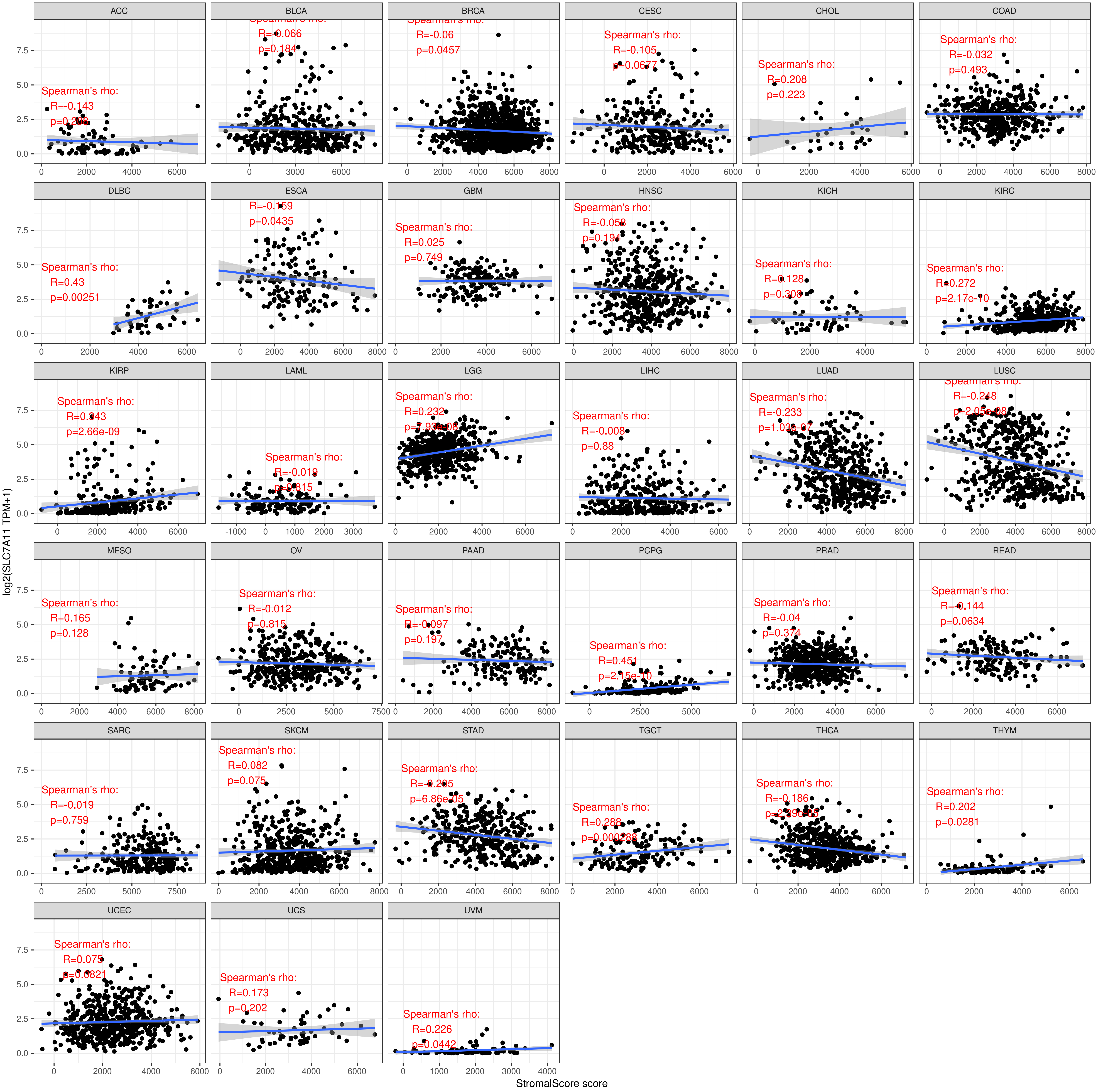

ACC
Spearman's rho:
R=-0.143
p=0.208
BLCA
R=-0.066
p=0.184
BRCA
R=-0.06
p=0.0457
CESC
Spearman's rho:
R=-0.105
p=0.0677
CHOL
Spearman's rho:
R=0.208
p=0.223
COAD
Spearman's rho:
R=-0.032
p=0.493
DLBC
Spearman's rho:
R=0.43
p=0.00251
ESCA
R=-0.159
p=0.0435
GBM
Spearman's rho:
R=0.025
p=0.749
HNSC
Spearman's rho:
R=-0.058
p=0.194
KICH
Spearman's rho:
R=0.128
p=0.308
KIRC
Spearman's rho:
R=0.272
p=2.17e-10
KIRP
Spearman's rho:
R=0.343
p=2.66e-09
LAML
Spearman's rho:
R=-0.019
p=0.815
LGG
Spearman's rho:
R=0.232
p=7.93e-08
LIHC
Spearman's rho:
R=-0.008
p=0.88
LUAD
Spearman's rho:
R=-0.233
p=1.03e-07
LUSC
R=-0.248
p=2.05e-08
MESO
Spearman's rho:
R=0.165
p=0.128
OV
Spearman's rho:
R=-0.012
p=0.815
PAAD
Spearman's rho:
R=-0.097
p=0.197
PCPG
Spearman's rho:
R=0.451
p=2.15e-10
PRAD
Spearman's rho:
R=-0.04
p=0.374
READ
Spearman's rho:
R=-0.144
p=0.0634
SARC
Spearman's rho:
R=-0.019
p=0.759
SKCM
Spearman's rho:
R=0.082
p=0.075
STAD
Spearman's rho:
R=-0.205
p=6.86e-05
TGCT
Spearman's rho:
R=0.288
p=0.000288
THCA
Spearman's rho:
R=-0.186
p=2.39e-05
THYM
Spearman's rho:
R=0.202
p=0.0281
UCEC
Spearman's rho:
R=0.075
p=0.0821
UCS
Spearman's rho:
R=0.173
p=0.202
UVM
Spearman's rho:
R=0.226
p=0.0442
log2(SLC7A11 TPM+1)
StromalScore score

Supplementary figure 5. The expression of SLC7A11 in STAD and PRAD was positively associated with neoantigen level.

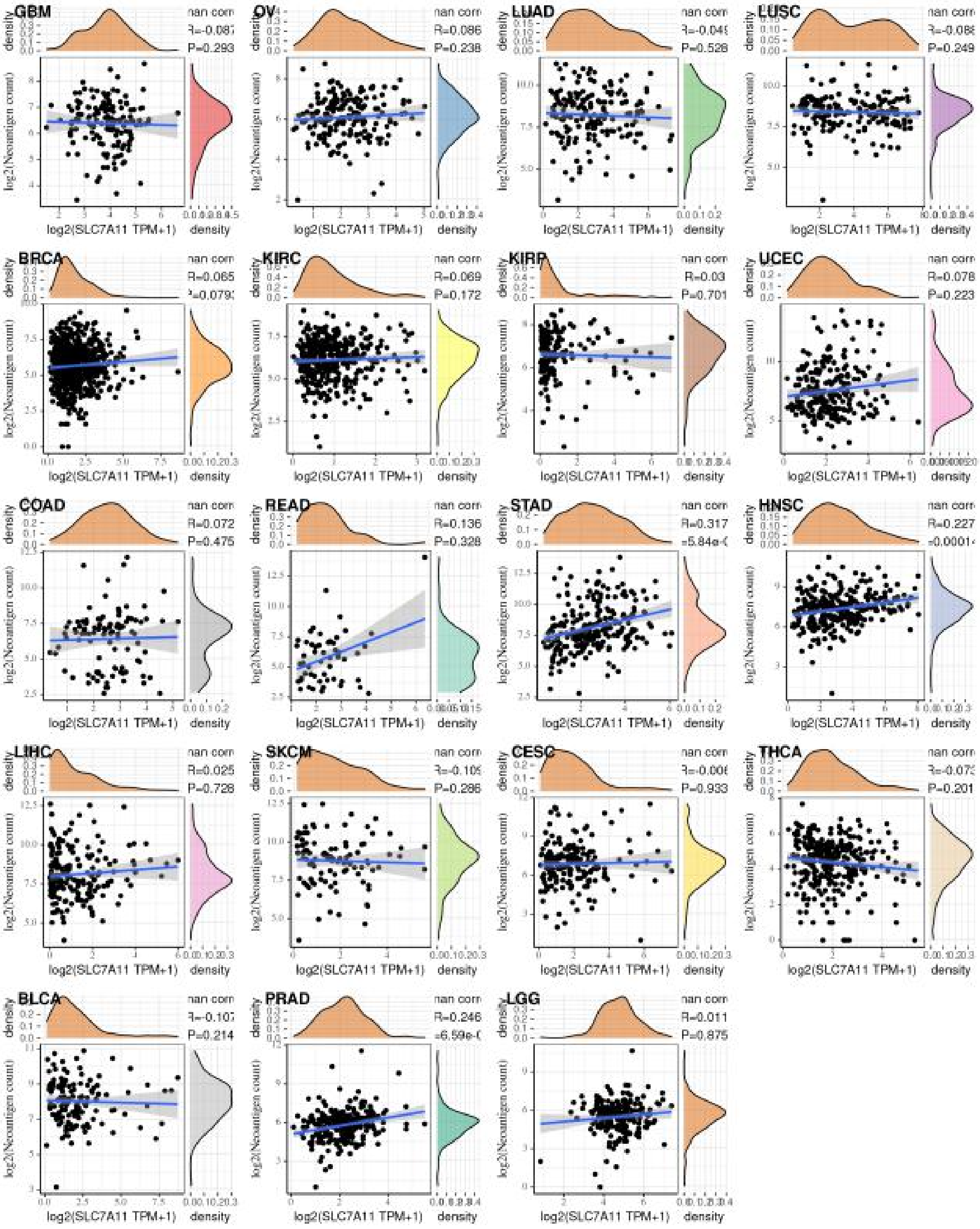

Supplementary figure 6. The mRNA and copy numbers of SCL7A11 were correlated with most molecules involved with MHC, chemokines and chemokines receptors.

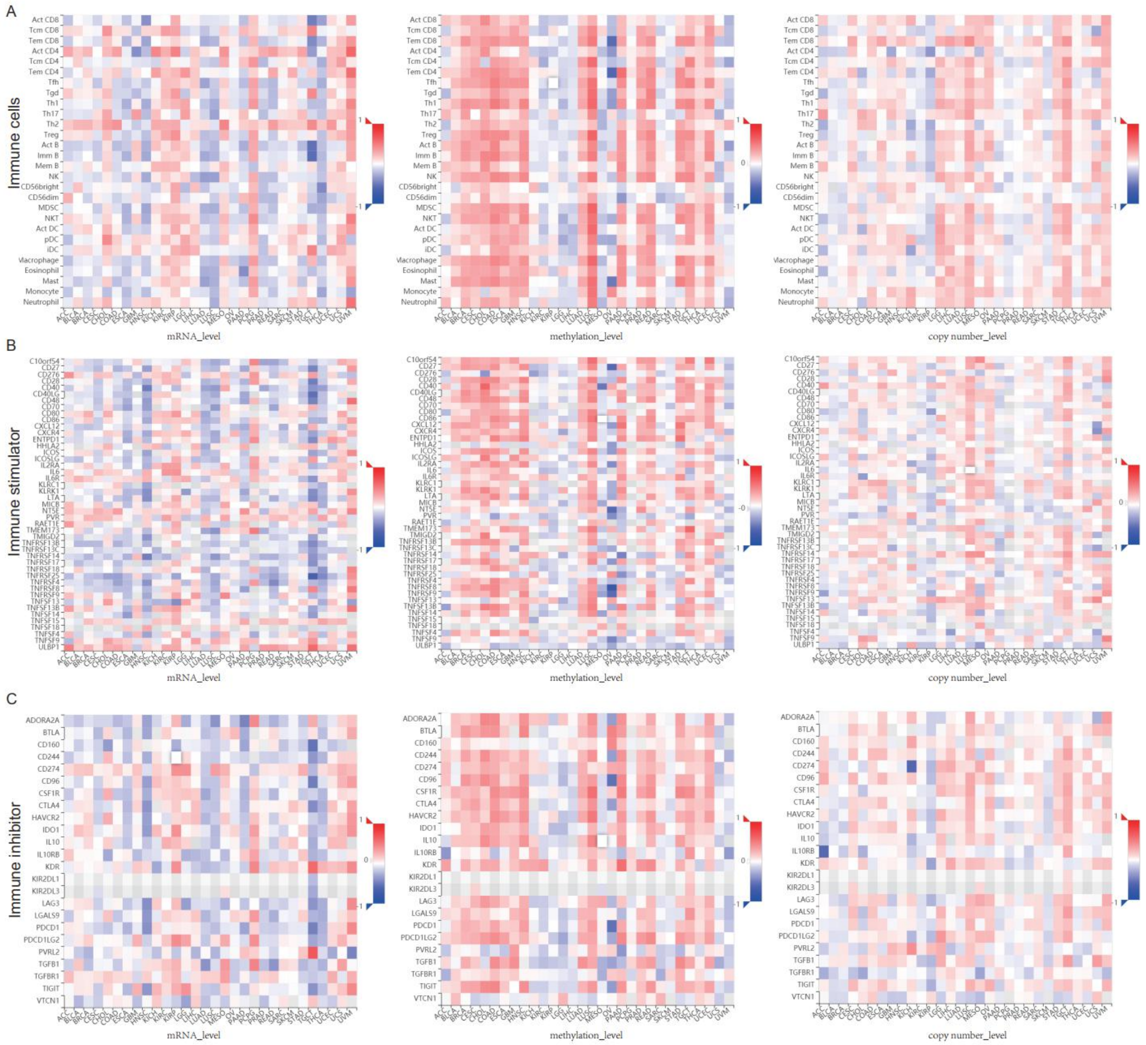

Supplement: Supplementary file 1 [file DataSheet3.PDF]
